# Impact of Helium Ions on Mouse Behavior, Memory, Microglia, and Blood Metabolome

**DOI:** 10.3390/biology15201819

**Published:** 2026-10-12

**Authors:** Svetlana S. Sorokina, Anton E. Malkov, Elizaveta A. Zamyatina, Alexander E. Shemyakov, Oleg V. Belov, Nelli R. Popova

**Affiliations:** 1 Institute of Theoretical and Experimental Biophysics of the Russian Academy of Sciences (RAS), Moscow Region, Pushchino 142290, Russia; malkovae@gmail.com (A.E.M.); sonyoru162@gmail.com (E.A.Z.); alshemyakov@yandex.ru (A.E.S.); nellipopovaran@gmail.com (N.R.P.); 2 P.N. Lebedev Physical Institute of the Russian Academy of Sciences, Moscow Region, Protvino 144281, Russia; 3 Joint Institute for Nuclear Research, Moscow Region, Dubna 141980, Russia; oleg.belov@jinr.int

**Keywords:** radiation therapy, space mission, helium ions, cognitive impairment, microglia, metabolites

## Abstract

The long-term space missions and helium ion (^4^He) radiotherapy require assessing of the consequences on the central nervous system. This study investigated the effects of total-body irradiation with 2 Gy helium ions on the brain of male Balb/c mice, focusing on cognitive function, microglial status, gene expression, and systemic metabolism. No statistically significant deficits in hippocampus-dependent behavior or spatial memory were detected. However, cellular and molecular alterations were detected. A decreased Iba1-positive cell density was observed at several post-irradiation time points, accompanied by morphological shifts towards a less ramified state. Gene expression analysis revealed a complex response, including a mixed neuroimmune activation profile, altered expression of redox-related genes, and downregulation of key defense genes. Metabolomics identified changes in 19 plasma metabolites consistent with alterations in energy and amino acid metabolism. Although 2 Gy did not manifest in overt cognitive impairment in the early delayed period, molecular and cellular alterations were detectable at 8 weeks, including decreased Iba1-positive cell density at several post-irradiation time points, changes in microglial morphology, altered expression of neuroimmune and redox-related genes, and systemic metabolic changes. These findings underscore the importance of multimodal assessment beyond behavioral testing in experimental models of helium-ion exposure. Because the exposure conditions used here differ from both the chronic mixed-field GCR environment and localized clinical helium-ion therapy, the results should be regarded as fundamental radiobiological observations rather than direct predictions of spaceflight or radiotherapy outcomes.

## Simple Summary

As humanity prepares for long-duration space missions and explores new methods for cancer treatment, it becomes essential to understand how exposure to helium radiation affects the brain. In this study, we exposed male mice to a single 2 Gy dose of helium ions. 5–8 weeks after exposure, we tested the animals’ memory and learning abilities, examined immune brain cells called microglia, measured metabolite changes in blood chemistry, and analyzed gene activity. No statistically significant differences in behavior and memory were detected under the conditions examined. However, further analysis revealed changes in the brain and blood: the number of Iba-1 microglia decreased, these cells changed their shape, and the levels of neuroimmune, redox-related, and DNA-repair genes and blood metabolites were altered. These observations may reflect ongoing biological changes, but they do not directly demonstrate impaired energy production or weakened cellular defenses, since metabolic flux and ATP production were not measured. These findings suggest that helium radiation can produce molecular and cellular changes even without detectable behavioral differences, and that longer-term studies are needed to determine whether these changes have functional consequences. This highlights the importance of using multiple testing methods to assess the effects of helium-ion exposure. Because the exposure conditions used here differ from both the chronic radiation environment of deep space and the localized delivery used in clinical helium-ion therapy, these findings should be regarded as fundamental observations that require confirmation in models that more closely reflect these scenarios.

## 1. Introduction

Research on the effects of helium ions on the central nervous system (CNS) is highly relevant. This is due to promising studies into unique, more effective methods for combating cancer [1,2], particularly in pediatrics [3,4], and the long-term planning of extended space exploration missions.

According to recent estimates, a journey to Mars will last from 1.5 to 3 years, making radiation exposure to the crew and associated impairments, particularly of the CNS, a significant concern [5,6,7]. It has been estimated that during deep space flights, one helium ion will pass through every cell of the body approximately once every three weeks. However, there is still a scarcity of research investigating its impact on CNS function [8,9,10]. With a typical mission duration of 180 days for the Mars transit, a similar period for the return, and an additional dose from the time spent on the Martian surface [11], the physical dose received from such a mission is expected to be substantially higher than that acquired by astronauts during six-month excursions on the International Space Station (ISS), approaching or even exceeding the current NASA dose limits for low Earth orbit missions [12]. Radiation protection limits for astronauts are administrative constraints rather than thresholds below which no biological effects occur. The current NASA career limit is 600 mSv, derived from the 3% REID for a 35-year-old female astronaut, with CNS-specific limits of 500 mGy over 30 days and 1000 mGy over 1 year.

To date, several authors have identified persistent, and sometimes progressive, radiation-induced deficits following exposure to several components of galactic cosmic radiation, such as protons, iron, silicon, titanium, and oxygen, either individually or in combination with each other [13,14,15,16,17,18,19]. It should be noted that a number of studies have investigated the effects of ions using head-localized irradiation. However, radiation exposure during deep space missions will not be confined to specific body regions. Furthermore, there is evidence that whole-body irradiation is more detrimental to cognitive function [20]. Cosmic radiation contains high-energy charged particles that deposit energy densely along their tracks, producing clustered DNA lesions rather than isolated damage. Because neurons are post-mitotic and cannot eliminate damaged cells through proliferation, they are particularly vulnerable to cumulative radiation-induced damage.

Understanding how radiation exposure affects cognitive function is crucial not only for astronauts on long-duration missions but also for patients undergoing cancer treatment. Whole-brain radiotherapy typically delivers a total dose of 30–40 Gy in 10–20 fractions, while higher total doses (e.g., 55–60 Gy) are used in focal or dose-escalated regimens rather than conventional whole-brain radiotherapy. This induces a wide spectrum of adverse effects, including impaired neurogenesis, damage to the blood–brain barrier, activation of glial cells, and infiltration of peripheral immune cells, which collectively can lead to long-term cognitive deficits [21]. In recent years, interest has grown in using heavier charged particles for cancer treatment. While proton therapy is currently the most common form of charged particle therapy, 14 centers worldwide now use carbon ions, and there is also interest in employing other ions in clinical practice, or even hybrid ion therapy [22]. Three decades after the end of the clinical program at the Lawrence Berkeley National Laboratory (LBNL) that used helium ion beams, the first patient was treated with scanned helium ions in July 2021 at the Heidelberg Ion Beam Therapy Center [23]. Over the past decade, several studies have been conducted to investigate helium ions in preparation for their potential clinical implementation [3,4,24]. Helium ions appear to be promising candidates for particle therapy due to their intermediate physical and radiobiological properties between proton and carbon ion beams [25,26].

It is now well-established that long-term changes in cognitive function following irradiation are accompanied by persistent impairments in hippocampal neurogenesis. Alongside alterations in the ratios of pools of maturing neurons, an elevated level of microglial activity is observed: there is an increase in the number of Iba1+ cells and GFAP+ astrocytes, along with changes in their morphology; the content of the synaptic proteins PSD-95 and MAP-2 increases due to disruptions in the Rac1-cofilin signaling pathway in the hippocampus and cerebral cortex [27,28]; tau protein levels rise in the cerebral cortex [29]; the number and complexity of myelinated axons decrease [30]; microvessel density changes; and mitochondrial function is impaired due to reduced activity of most proteins necessary for ATP production [27]. A recent study suggests that changes in neurogenesis and neuroinflammation may resolve earlier than the behavioral alterations in animals [31], while other reports suggest that an increased number of activated microglial cells in the hippocampus and a decreased number of astrocytes persist for up to 24 months, a time point at which behavioral deficits are no longer detected [32].

It should be emphasized that the experimental exposure used in the present study differs substantially from both translational contexts. During deep-space missions, crew members are exposed chronically to a mixed field of galactic cosmic rays at low dose rates, over periods of months to years. Conversely, clinical helium-ion radiotherapy delivers highly localized, treatment-planned doses at high dose rates, with steep dose gradients and sparing of surrounding healthy tissue. The 2 Gy whole-body exposure used here does not directly represent either scenario; rather, it serves as an experimental model to probe cellular and molecular responses to helium ions at a clinically relevant fractional dose.

In this study, we investigated the effects of a 2 Gy dose of helium ions delivered by whole-body irradiation in mice on behavior, memory, and microglial morphology at different time points post-irradiation. Microglial changes may play a critical role in the development of cognitive deficits. To assess the early delayed effects of the ions, we evaluated changes in blood metabolites in the irradiated mice using 1H NMR spectroscopy and measured the expression levels of a number of key genes in the brain tissue using PCR 8 weeks after exposure.

We believe it is important to combine gene expression analysis and histochemical methods for assessing delayed radiation-induced impairments with NMR-based metabolomic profiling, which allows the simultaneous detection of a broad range of low-molecular-weight metabolites in biological fluids. Radiation can alter the ratio and concentration of endogenous metabolites either through direct chemical reactions or by binding to key enzymes or nucleic acids that control metabolism. If these disruptions are sufficiently extensive, additional toxic effects can arise.

## 2. Materials and Methods

Animals. Male Balb/c mice (*n* = 40, 8 weeks old, 20–24 g, “Stolbovaya” breeding station, Russia) were used in the experiment. All animals were individually identified by applying a 3–5% aqueous solution of picric acid to a small patch of fur on the back region, producing a yellow stain, and were randomly assigned to experimental groups using a computer-generated random number sequence (GraphPad Randomizer 11.1.0) to minimize selection bias.

The animals were housed in standard polycarbonate cages (five per cage) with autoclaved wood chip bedding in a climate-controlled facility of the Institute of Theoretical and Experimental Biophysics (ITEB), Russian Academy of Sciences (Pushchino). The environment was maintained at 22 ± 2 °C with a 12/12 h light/dark cycle (lights on at 7:00 a.m.). Food (complete extruded diet, Provimi LLC, Moscow, Russia) and filtered tap water were provided *ad libitum*. Cage enrichment (nesting material) was provided to promote natural behavior. The sample size for the behavioral cohort (n = 10 per group) was chosen based on our preliminary experiments, which demonstrated that this number provides sufficient statistical power (≥80%) to detect a 20% difference in spatial memory performance between groups, with a standard deviation of approximately 15%. To avoid circadian rhythm interference, all behavioral tests were conducted between 9:00 a.m. and 2:00 p.m. The experimenter performing the behavioral scoring and metabolomic analysis was blinded to the group allocation; the codes were revealed only after the primary statistical analysis. Animals were monitored daily for signs of distress or weight loss (>15% of initial weight), which served as humane endpoints. At the conclusion of the experiment, mice were euthanized by cervical dislocation under deep isoflurane anesthesia to minimize suffering.

The experimental protocol complied with the ethical standards governing the use of laboratory animals (European Convention for the Protection of Vertebrate Animals used for Experimental and other Scientific Purposes: ETS No. 123, 18 March 1986, Strasbourg; “Good Laboratory Practice Rules”, approved by Order No. 199n of the Ministry of Health of the Russian Federation on 1 April 2016; ITEB Ethics Committee). The animal study protocol was approved by the Institutional Ethics Committee of Institute of Theoretical and Experimental Biophysics (ITEB RAS) (protocol № 03/2026 from 25 February 2026) for studies involving animals.

**Figure 1:**
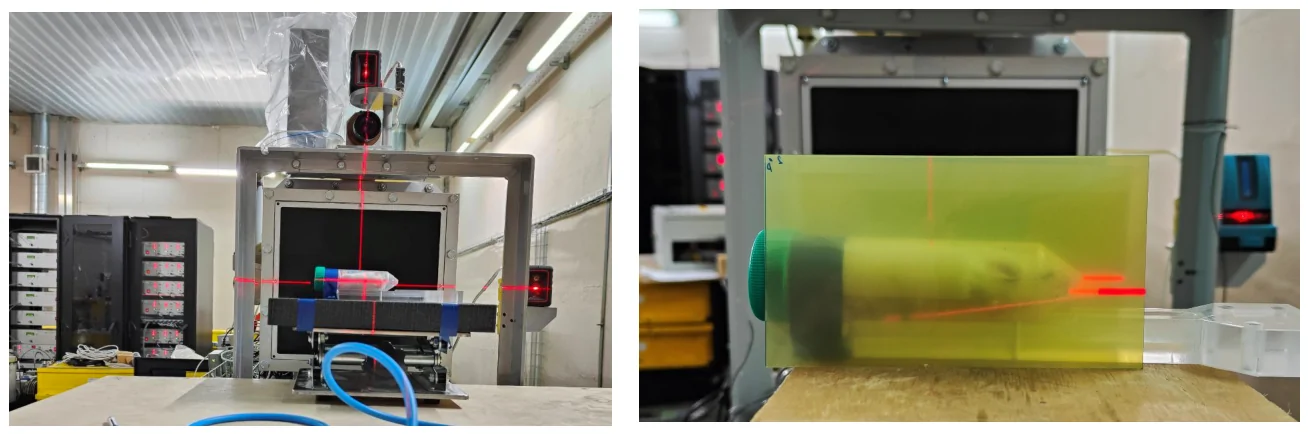
Setup for animal positioning at the Prometheus proton therapy facility (JSC “Protom”, Protvino).

Irradiation. Accelerated helium ion irradiation was performed at the synchrotron of the “Prometheus” complex (JSC “Protom”, Protvino, Russia) (Figure 1). The animals were positioned using vials. Irradiation was performed sequentially on one animal at a time using the beam scanning method over a specified volume. A dose of 2 Gy (with a dose delivery uncertainty of ±10%) was delivered with a helium ion energy of 320 MeV (80 MeV/nucleon). A particle energy of 320 MeV corresponds to a range of approximately 50 mm in water. This helium ion energy is characterized by an extremely sharp energy deposition at the end of the particle range, which does not allow the Bragg peak to be used for animal irradiation. Therefore, the mice were irradiated in compact vials (without anesthesia). The maximum width of the vial was 28 mm. To minimize dose non-uniformity along the depth, the vial containing the animal was positioned perpendicular to the beam. The irradiation field size was 100 × 50 mm. Dosimetric measurements performed using a PTW PinPoint 3D ionization chamber before and after the vials with animals showed a variation in absorbed dose of less than 10%, indicating a reasonably uniform dose distribution along the depth of the mouse’s body. Similar results were obtained regarding the uniformity of the lateral dose distribution through the combined analysis of EBT3 dosimetric films irradiated in the same positions as the ionization chamber. According to ASTAR (NIST), the LET value for this particle energy is approximately 3.4 keV/µm. A specific feature of synchrotrons is their cyclic operation; in this mode, the duration of one cycle was 2.5 s. The average duration of one irradiation session was 5 min and 20 s. The non-irradiated (control) group of animals underwent the same procedures as the experimental group, but with the accelerator turned off.

Behavioral tests. Five–seven weeks after irradiation, a series of behavioral tests were conducted to assess general activity, anxiety levels, spatial learning, and short-term and long-term hippocampus-dependent memory. The tests were performed using standardized protocols and certified equipment (LLC “NPK Open Science”). The behavioral battery included the open field test, the elevated plus maze, the light-dark box test, and the Barnes maze.

Open Field test (OF, 4 min): An assay designed to evaluate general locomotor activity, exploratory behavior, and anxiety-like states. The mice were placed in a novel, brightly lit, and enclosed square arena. Parameters measured typically include average velocity, total distance traveled (horizontal activity), number of freezing, grooming and rearing acts (vertical activity), frequency and time spent in the anxiolytic center zone versus the thigmotactic periphery.

Elevated Plus Maze (EPM, 5 min): A widely used unconditioned test for anxiety-like behavior based on the conflict between the innate curiosity of rodents to explore a novel environment and their aversion to open, elevated spaces. The apparatus consists of a plus-shaped maze elevated above the floor, with two open arms (without walls) and two enclosed arms (with high walls). The primary indices are the total distance moved and velocity, time until first entry, time spent in, and number of entries into the open arms relative to the closed arm exploration.

Light-Dark Box test (LDB, 5 min): A conflict-based test measuring anxiety-like behavior and exploratory drive, utilizing the natural preference of rodents for dark, enclosed spaces over brightly lit, open areas. The apparatus comprises two compartments: one enclosed dark compartment and an illuminated open area connected by a small opening. Key metrics include velocity and distance moved, latency to first enter the light compartment, time spent in the light compartment, and the number of transitions between compartments.

Barnes Maze: A visuo-spatial learning and memory task used to assess hippocampal-dependent spatial reference memory and cognitive flexibility. The subject must learn and remember the location of a hidden escape box located under one of many holes positioned around the perimeter of a large, circular, brightly lit platform. Primary measures are latency to find the escape box and number of errors (investigation of incorrect holes) across training (3 trials per day for 3 min) and probe trials (test 1 and test 2 by 2 min). All tests were accompanied by automated video tracking of the mice using specialized software. An animal was considered to have completed the task if it entered the escape box within the 180 s trial. Animals that did not enter the escape box within this period were assigned a latency of 180 s (the maximum trial duration).

Microglia. Brains of five groups of animals (n = 20) each with different time after irradiation (control n = 5, 1 day n = 4, 1 week n = 5, 1 month (4 weeks) n = 3, 2 months (8 weeks) n = 3) were stained for the microglial marker Iba-1. The control group consisted of five animals: two animals were euthanized at the 1-day time point, and one animal was euthanized at each of the 1-week, 1-month, and 2-month time points. Thus, the pooled control group included animals sampled across all post-irradiation intervals; however, a separate control cohort was not available for each time point. Animals were comparable in terms of the main experimental characteristics, including age, sex, and experimental conditions. The use of a pooled control group should be considered a limitation because it does not fully exclude time-dependent variability in control microglial parameters. Free-floating slices (35 µm) from perfused animals were incubated with Triton X (0.3%) in PBS three times for 5 min, followed by blocking solution (BSA 1%, Triton X 0.3% in PBS) for 2 h. Then, the primary antibody rabbit anti-Iba-1 (1:1000; Wako, Japan) was added and slices were incubated overnight at 4 °C. The next day, the secondary antibody, goat anti-rabbit (1:1000; Alexa Fluor 488, ThermoFisher, Waltham, MA, USA), was added for 2 h. After washout in PBS with 0.3% TritonX-100, slices were mounted on gelatinized coverslips in Fluoromount media (Sigma-Aldrich, St. Louis, MO, USA). Immunostaining was analyzed under a Nikon E200 fluorescence microscope (Nikon, Tokyo, Japan). In order to make a proper comparison, equivalent regions of the dorsal hippocampal CA1 field (from −1.5 to −2.8 relative to bregma) from both hemispheres were chosen for all the groups. Photomicrographs using 10× (0.25 of numerical aperture) and 40× for morphological parameter evaluation of stained fluorescence were quantified with the ImageJ software 1.54 (NIH, Bethesda, MD, USA). For cell density analysis, one photomicrograph containing the central part of the CA1 field of the dorsal hippocampus was acquired from each analyzed section at 40× magnification. Each image covered the full microscope field of view and corresponded to an area of approximately 0.173 mm^2^. Iba1-positive cells within the entire field of view were counted, and microglial cell density was expressed as the number of Iba1-positive cells per square millimeter (cells/mm^2^). The following morphological parameters of Iba-1+ cells were assessed using custom macros: cell perimeter, cell area, circularity (4*π*(area/perimeter^^2^)), aspect ratio (major axis/minor axis), solidity (area/convex hull area). Image acquisition and quantitative analysis were performed blind to the experimental group.

Microglial immunohistochemical data were analyzed using a hierarchical mixed-effects approach to account for the nested structure of the dataset, in which individual cells were sampled from sections, and multiple sections were obtained from the same animal. The primary experimental unit was the animal rather than the individual cell. To avoid pseudoreplication, individual cells were not entered into the statistical model as independent observations. For each section, microglial cell number was calculated for the density analysis, whereas the mean value of each morphological parameter was calculated across all analyzed microglial cells within that section. Thus, section-level observations were nested within animals.

For each parameter, a linear mixed-effects model was fitted with experimental group as a fixed effect and animal identity as a random intercept. The control group was specified as the reference category. This model accounts for the non-independence of sections obtained from the same animal while allowing the number of sections analyzed per animal to vary.

For each contrast, the estimated between-group difference (estimate), standard error, 95% confidence interval (CI), and two-sided *p* value were calculated. Because four planned comparisons with the control group were performed for each morphological parameter, *p* values were adjusted using the Holm procedure within each parameter. Statistical significance was defined as an adjusted *p* < 0.05. Given the relatively small number of animals per group, effect estimates and confidence intervals were considered alongside *p* values rather than relying on statistical significance alone.

NMR Spectroscopy. Blood Sampling. Eight weeks after exposure, one week after the completion of behavioral testing, animals were anesthetized with isoflurane and euthanized by decapitation, followed by terminal blood sampling. Blood from 5 control and 5 irradiated animals was collected in tubes containing heparin, centrifuged (1600 *g*, 4 °C, 15 min), and the resulting plasma was frozen at −20 °C until NMR analysis. Samples underwent a single freeze–thaw cycle before NMR analysis.

Mouse plasma samples (about 100 μL each) stored at −20 °C were thawed, mixed with 40 μL of 1 M phosphate buffer (pH 6.9) prepared with deuterated water D2O (99.9% deuterated, Sigma, USA) and containing 4 mM maleic acid (MA) as an internal standard, and the sample volume was then adjusted to 580 μL with distilled water.

The sample was vigorously mixed for 30 s, centrifuged at 15,000 g for 5 min, and placed in the NMR tube (5 mm in diameter). NMR spectra were recorded at 298 K on an AVANCE III 600 spectrometer (Bruker, Bremen, Germany) (Center for Collective Use, Institute of Theoretical and Experimental Biophysics, Pushchino, Russia), with an operating proton frequency of 598.95 MHz. 1D NMR spectra were recorded on a spectrometer using standard pulse sequences from the Bruker pulse sequence library. All measurements were performed at 298 K (25 °C). The signal from water protons was suppressed by a presaturation method using the 1D CPMG pulse sequence from the Bruker library; the relaxation delay time was 5 s; the sweep was 10 ppm. The number of accumulations was 256 scans (t ~35 min). The chemical shifts were internally referenced to the α-glucose anomeric proton doublet resonance at 5.24 ppm. The maleic acid signal (~6.05 ppm) was used as an internal standard; however, absolute metabolite concentrations were not determined in this study. No quantitative or semi-quantitative per-animal metabolite data were obtained. Metabolite levels were assessed only as relative signal intensities in group-averaged spectra, as described below. Spectra were accumulated and processed using TOPSPIN (Bruker, version 2.1). Metabolites were identified using AMIX (Bruker, version 3.9.7).

Untargeted analysis of NMR spectroscopy data obtained in the experiment was carried out using the R programming language (v. 4.3.0) in the RStudio environment (v. 2023.06.0 + 421) and the metabom8 library [33] with the open-source code available at GitHub (https://github.com/tkimhofer/metabom8, v. 1.0.0, accessed on 22 June 2026). The spectra were calibrated based on the glucose doublet located in the 5.15–5.30 ppm range. They were also subjected to baseline correction, PQN normalization [34], and removal of the up- and downfield ranges containing noises and the TSP signal; the 4.50–5.00 ppm range containing residual signals of water protons was also removed. Principal component analysis (PCA) was used to assess intergroup differences and the contributions of individual metabolites to these differences. The above method of multivariate data analysis was implemented using the metabom8 library, which contains appropriate functions for their execution.

Quantitative Real-Time PCR (qPCR). The expression levels of the following genes were assessed by quantitative real-time PCR: CCL2, CD163, CD206, TLR4, CX3CR1, BCL2, RAD51, ATM, HO-1, SOD1, SOD2, and CAT. Total RNA was extracted from whole-brain tissue using the HiPure Total RNA Plus Kit (Magen, Guangzhou, China) according to the manufacturer’s standard protocol. Three biological replicates per group (n = 3) were analyzed. The concentration and purity of the isolated RNA were measured using a DS-11 FX+ spectrophotometer (DeNovix, Wilmington, DE, USA). RNA quality criteria included the A260/A280 and A260/A230 ratios; only samples with A260/A280 within 1.8–2.1 and A260/A230 ≥ 1.8 were used for reverse transcription. Reverse transcription was performed using the MMLV RT kit (Evrogen, Moscow, Russia) according to the standard protocol, with 1600 ng of total RNA used as input. TBP was used as the reference gene for all samples. The stability of the reference gene was verified using the online tool RefFinder. Gene-specific primers used in the experiment are listed in Table 1. The qPCR reaction was carried out using the qPCRmix-HS SYBR+LowROX mix (Evrogen, Russia) on a Gentier 96E detection system (Tianlong, Anshun, China). Amplification specificity was confirmed by melt-curve analysis, which showed a single peak for each primer pair. No-template controls were included in each qPCR run and were negative. Primer amplification efficiency was assessed using standard curves generated from serial dilutions of pooled cDNA; efficiencies were 98.5–105.2% with R^2^ > 0.995. Relative gene expression was calculated using the 2^−ΔΔCt^ method.

Statistical analysis. All calculations were performed using GraphPad Prism, 10.4.0. Differences between groups were assessed for significance using the Mann–Whitney U test, with a significance level set at *p* < 0.05. Statistical comparison of the learning curves was performed using analysis of variance (ANOVA) in the IgorPro 8 statistical software package. Repeated latency measurements for Barnes maze were analyzed using generalized estimating equations (GEE) with a Gaussian working distribution, an exchangeable within-animal correlation structure, and robust standard errors. The model included experimental group (control or helium-ion irradiation), trial number, and the group × trial interaction as fixed effects. Animal identity was used as the clustering variable. Trial number was centered at its mean before model fitting. The group × trial interaction was considered the primary test of whether learning trajectories differed between groups. The results are reported as regression estimates, robust standard errors, 95% confidence intervals, and two-sided *p* values. For microglia analysis, the group comparisons were performed using linear mixed-effects models with experimental group as a fixed effect and animal identity as a random intercept, thereby accounting for repeated section-level observations from the same animal. *p* values were adjusted for the four planned comparisons with controls using the Holm procedure. For qPCR data, three biological replicates (n = 3) per group were analyzed. Between-group differences were assessed using the Mann–Whitney U test. To account for multiple comparisons across the 12 genes analyzed, *p*-values were adjusted using the Benjamini–Hochberg false discovery rate (FDR) procedure. A gene was considered differentially expressed when the FDR-adjusted q-value was <0.05. Data are presented as mean ± standard deviation (SD) across biological replicates.

## 3. Results

**Figure 2:**
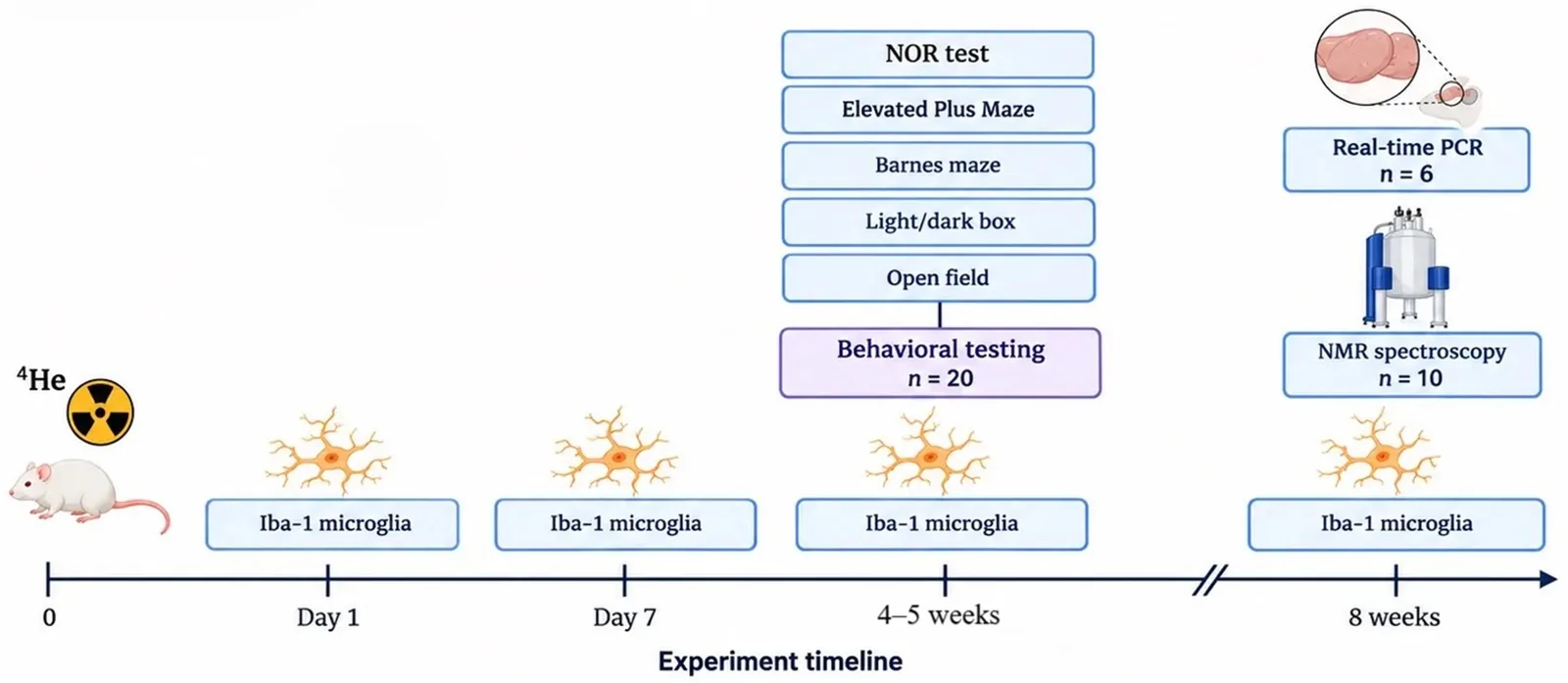
Timeline of experimental design: investigation of the helium ion effect on mouse behavior, memory, microglia, and blood metabolome in short and delay periods.

A schematic of the experimental design is shown in Figure 2.

### 3.1. Behavior Results

#### 3.1.1. Open Field Test

**Figure 3:**
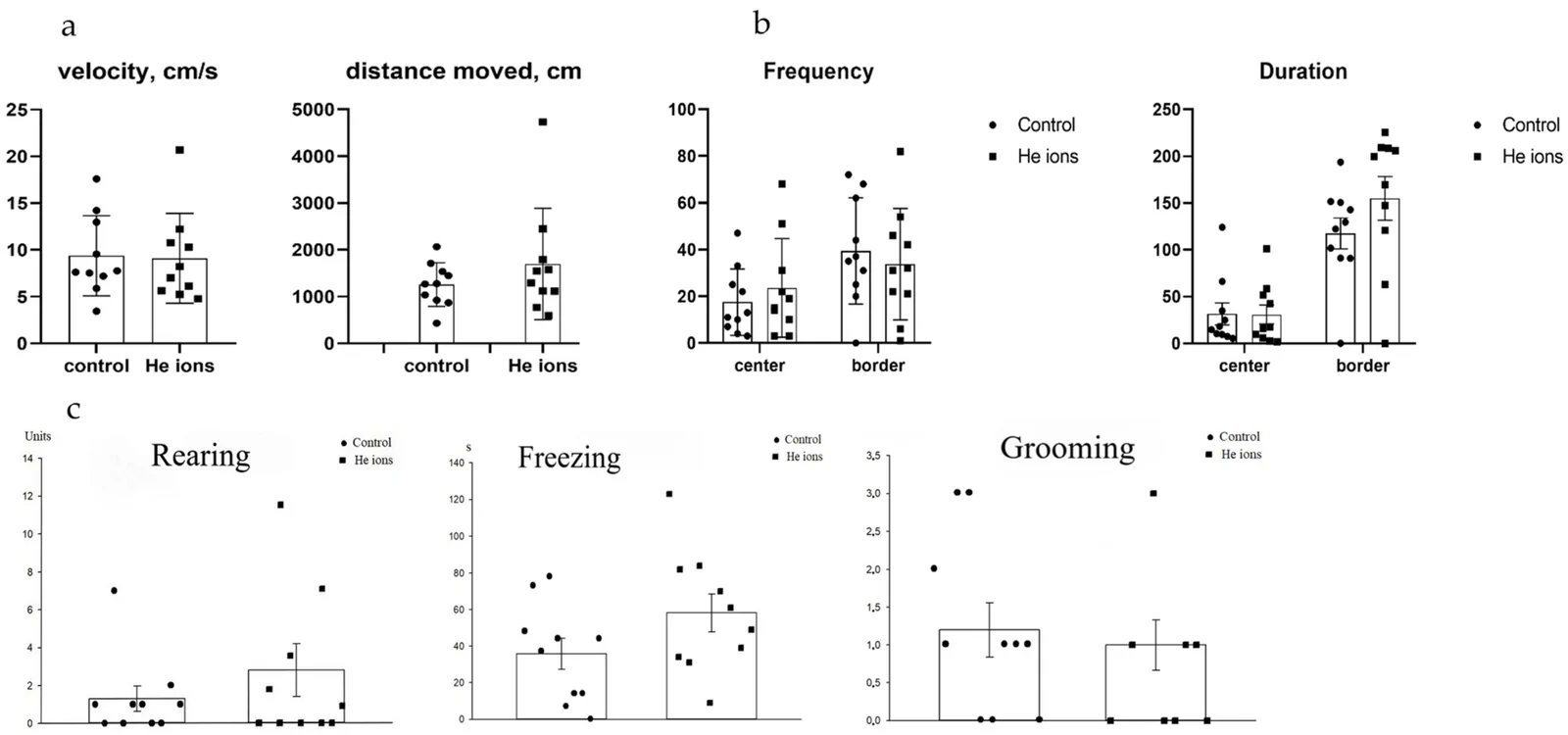
Changes in exploratory behavior in the “open field” test in mice exposed to helium ions at a dose of 2 Gy: velocity and total distance moved (**a**); exit frequency and total duration of stay (**b**) in the center and at the border (periphery) of the setup; number of grooming acts, freezing episodes and rearing acts (**c**). Data are shown as the mean ± SD (n = 10 animals per group).

In the square open field setup, standard indicators were recorded over a 4 min period: total distance traveled, frequency of entries into and time spent by the animal in the central area versus the periphery (walls), as well as the average speed of movement within the apparatus. Analysis of the results did not reveal any statistically significant changes. As can be seen from Figure 3a, the irradiated and control groups of mice exhibited similar levels of locomotor activity. Furthermore, regarding the frequency of entries into different sectors of the apparatus and the total time spent in them, Figure 3b also shows no significant differences between the control and experimental groups. It is known from the literature that Balb/c mice generally exhibit relatively low activity in the open field test [35]. Analysis of the number of rearing, freezing episodes, and grooming acts also revealed no significant differences between the experimental and control groups (Figure 3c).

According to a number of authors, this mouse strain is characterized by a relatively high level of anxiety, which is manifested, in particular, by a high frequency of urination and defecation in the testing apparatus [36,37]. In our assessment, the average number of boluses in the control and irradiated animal groups during the open field (OF) test was 1.7 ± 0.49 and 2.0 ± 0.63, respectively, while the number of urination acts was 0.5 and 0.2, respectively.

Thus, overall, the results of the OF test demonstrate a predominance of exploratory behavior over defensive behavior. No statistically significant psycho-emotional state impairment was detected under the experimental conditions examined 7 weeks after exposure.

#### 3.1.2. Barnes Maze

**Figure 4:**
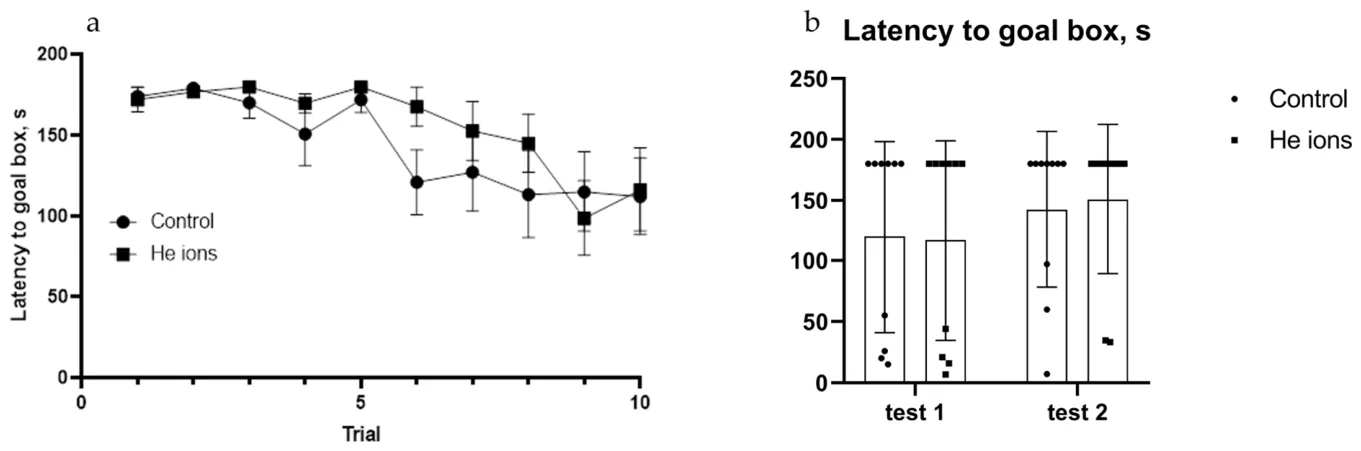
The effect of 2 Gy of helium ions on spatial learning and memory performance in mice in the Barnes maze test: (**a**) Barnes-maze learning curves in control and helium-ion-irradiated mice. Escape latency is shown across 10 training trials. Points represent group means, and error bars represent SEM; control, n = 10; helium-ion irradiation, n = 10. Latency decreased across trials (*p* = 0.001), whereas the group effect (*p* = 0.369) and group × trial interaction (*p* = 0.862) were not statistically significant. The maximum permitted latency was 180 s; (**b**) testing session—probe 1, testing animals in the maze on day 3 after learning; probe 2, testing animals in the maze on day 9 after learning.

Barnes-maze learning curves were analyzed in 10 control and 10 helium-ion-irradiated mice across 10 training trials (Figure 4a). Escape latency decreased significantly across trials in the control group (estimate = −8.44 s per trial, 95% CI [−13.59, −3.29], *p* = 0.001), indicating improvement in task performance across repeated trials. At the mean trial number, the estimated latency in the helium-ion group was 12.15 s higher than in the control group; however, this difference was not statistically significant (95% CI [−14.33, 38.63], *p* = 0.369).

The group × trial interaction was not statistically significant (estimate = 0.64 s per trial, 95% CI [−6.61, 7.89], *p* = 0.862). Mean escape latency decreased from 174.59 s during trials 1–3 to 113.70 s during trials 8–10 in the control group and from 176.44 s to 120.23 s, respectively, in the helium-ion group. However, 69.7% of the control observations and 79.0% of the irradiated-group observations reached the maximum permitted latency of 180 s.

During the long-term memory test (Figure 4b) on day 3 (test 1) and day 9 (test 2) after training, no significant differences were detected in latency to reach the goal box (n = 10 per group). In both groups, 50% of the animals (5 out of 10 per group) completed the task within the 180 s trial. The remaining animals were assigned the maximum latency of 180 s. Importantly, the proportion of non-completing animals was identical between groups, and no statistically significant difference in task completion was detected. All data are presented as mean and SD.

#### 3.1.3. Elevated Plus Maze (EPM) Test

**Figure 5:**
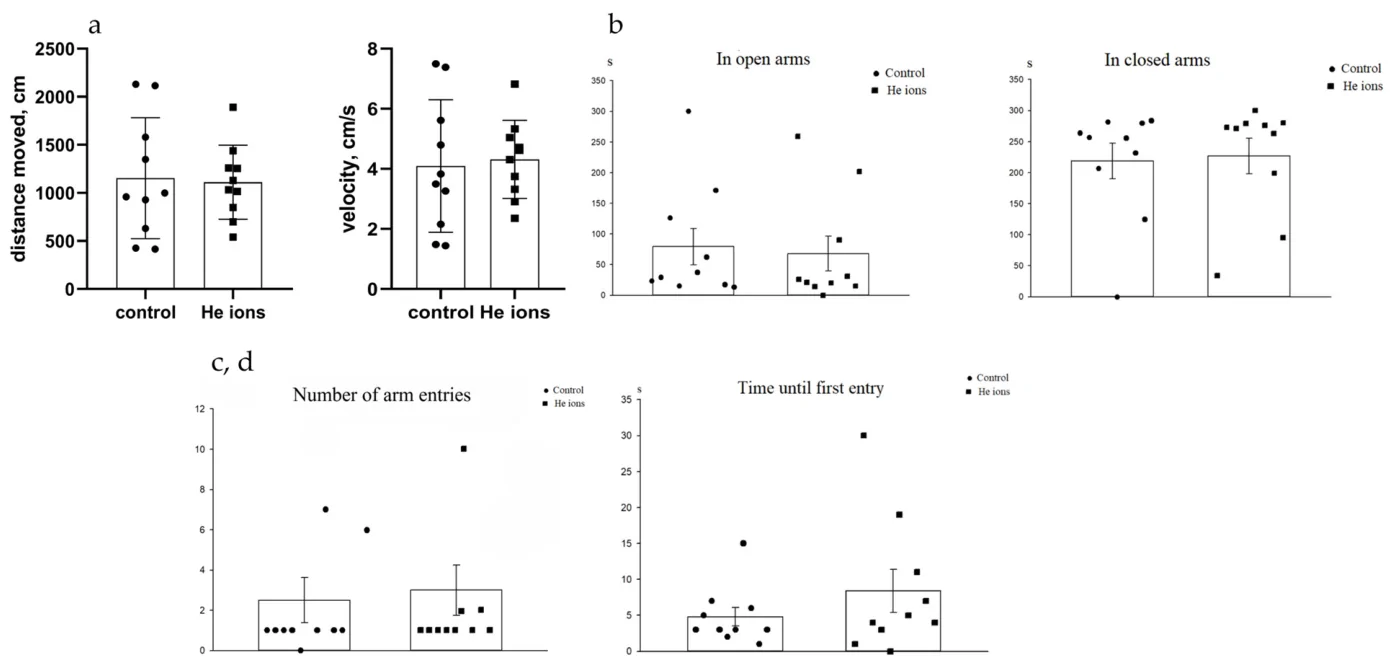
Changes in exploratory behavior in the EPM test in mice exposed to helium ions at dose of 2 Gy: distance moved and velocity (**a**); time spent by animals in the open and closed arms (**b**); number of arm entries (**c**) and time until first entry (**d**). Data are shown as the mean ± SD (n = 10 animals per group).

The EPM test assesses locomotor activity, the speed of orienting responses, and the severity of fear- and anxiety-like emotional responses. The less pronounced the anxiety response and the phobic component of the emotional status in an animal, the more time it spends in the open arms and the less frequently it enters the closed arms during the observation period. As shown in Figure 5a, irradiation with 2 Gy of helium ions did not affect the overall locomotor activity of the mice. Furthermore, mice from both experimental groups showed an equal preference for spending time in the closed arms (Figure 5b) and made a similar number of transitions between the arms (Figure 5c). It should be noted that the time until the first entry into the closed arms increased in the group of irradiated mice; however, this difference was not statistically significant (Figure 5d). According to our data, the average number of boluses in the control and irradiated animal groups during the EPM test was 2.5 ± 0.54 and 3.6 ± 0.81, respectively, while the number of urination acts was 0.2 and 0.3, respectively.

#### 3.1.4. Light-Dark Box (LDB) Test

**Figure 6:**
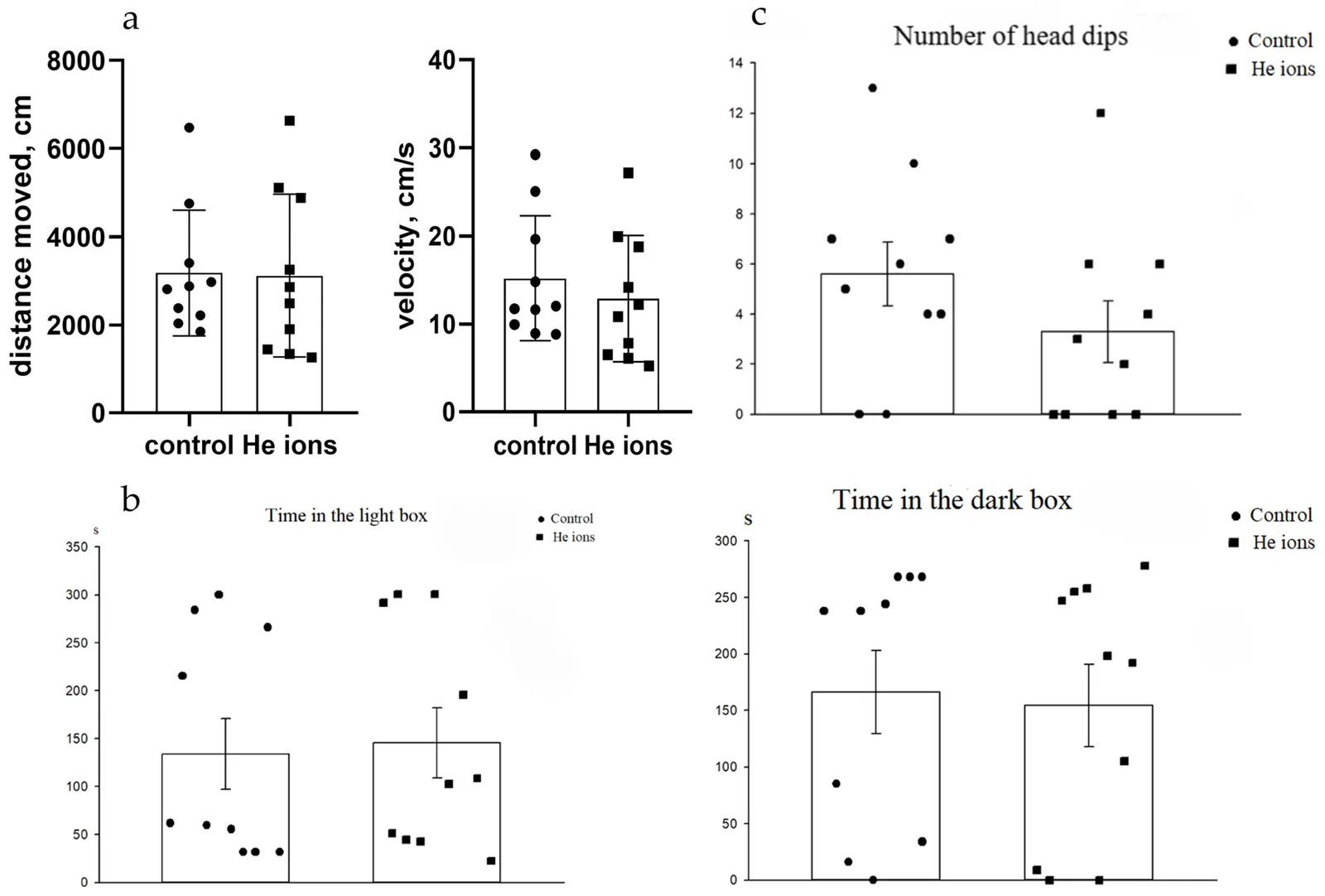
Changes in exploratory behavior in the LDB test in mice exposed to helium ions at a dose of 2 Gy: distance moved and velocity (**a**); time spent by animals in the light and dark box (**b**); number of head dips (**c**). Data are shown as the mean ± SD (n = 10 animals per group).

No differences in general locomotor activity were found between the groups in the LDB test either (Figure 6a). Furthermore, neither the time spent in the light and dark compartments (Figure 6b) nor the risk assessment behavior in mice irradiated with helium ions differed from the control group (Figure 6c). The average number of boluses in the control and irradiated animal groups during the LDB test was 1.1 ± 0.41 and 2.5 ± 0.83, respectively, while the number of urination acts was 0 and 0.1, respectively.

### 3.2. Microglia

A total of 569 tissue sections and 56.785 microglial cells were analyzed. The mean number of analyzed sections per animal was 45.4 ± 18.3 in the control group, 24.8 ± 6.7 in the 1-day group, 19.6 ± 18.2 in the 1-week group, 30.7 ± 14.0 in the 1-month (4 weeks) group, and 17.7 ± 3.1 in the 2-month (8 weeks) group.

Because sections and cells were nested within animals, group differences were evaluated using linear mixed-effects models with animal identity included as a random intercept. This approach prevented the large number of individual cells and sections from being treated as independent biological replicates.

**Figure 7:**
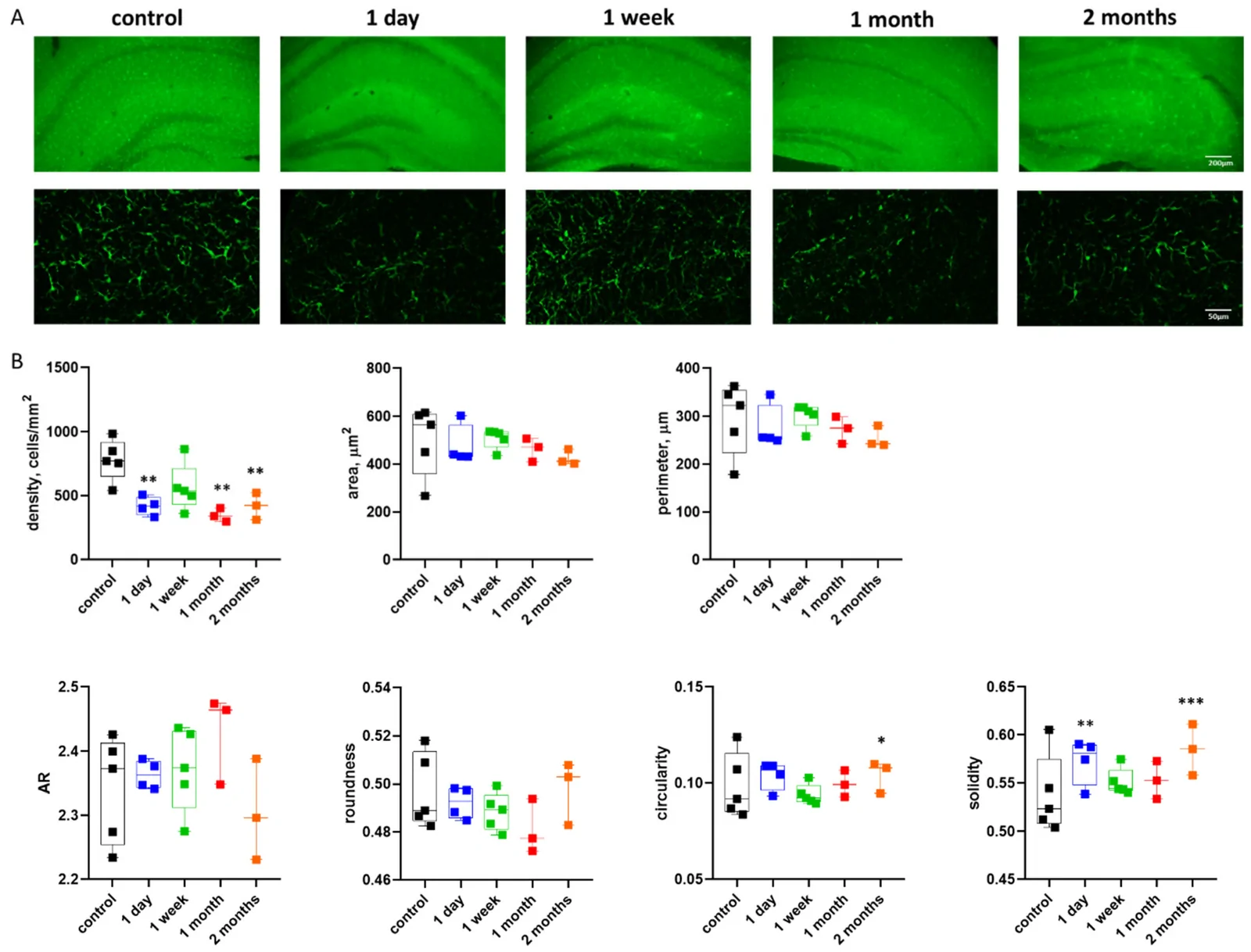
Changes in the morphology of microglia at different times after irradiation: (**A**) photographs of brain sections with immunohistochemical staining for Iba-1, magnification 10× and 40×; (**B**) averaged morphological parameters at different times after irradiation. (Black) control; (blue) 1 day after irradiation; (green) after 1 week; (red) after 1 month; (orange) after 2 months, * *p* < 0.05, ** *p* < 0.01, *** *p* < 0.001. Each point represents one animal and shows the mean of its section-level measurements. Group differences were tested using linear mixed-effects models fitted to section-level data, with animal identity included as a random intercept. Box plots represent group means ± min/max.

Quantitative analysis of microglial morphology revealed statistically significant, albeit variably expressed, alterations across multiple structural parameters following irradiation. Although all changes were significant, their magnitude varied considerably, indicating that some morphological shifts were more subtle than others (Figure 7A,B).

Cell Density. The mean densities of Iba1-positive cells were 781.99 cells/mm^2^ in the control group, 419.54 cells/mm^2^ at 1 day, 564.96 cells/mm^2^ at 1 week, 344.51 cells/mm^2^ at 1 month, and 417.17 cells/mm^2^ at 2 months after irradiation. Compared with the control group, the density of Iba1-positive cells was significantly lower at 1 day after irradiation (estimate = −397.34 cells/mm^2^, 95% CI [−655.49, −139.31], adjusted *p* = 0.004), at 1 month (estimate = −451.39 cells/mm^2^, 95% CI [−721.39, −181.50], adjusted *p* = 0.003), and at 2 months (estimate = −397.05 cells/mm^2^, 95% CI [−668.21, −126.01], adjusted *p* = 0.007). No significant difference in Iba1-positive cell density was detected between the 1-week and control groups (estimate = −105.84 cells/mm^2^, 95% CI [−343.93, 132.37], adjusted *p* = 0.377). Although the difference in cell density at 1 week did not reach statistical significance, the pattern across time points indicates a sustained reduction in microglial abundance. The underlying mechanisms (e.g., altered survival, proliferation, or phenotype-dependent detectability) were not directly assessed in this study.

Cell Morphology. For cell morphology, no statistically significant differences from K remained after Holm correction for area. Although 2 months group showed a relatively large reduction in cell area compared with K, this difference did not reach the predefined significance threshold (*p* = 0.065 after adjustment).

No adjusted significant differences were detected for perimeter, aspect ratio, or roundness. A significant increase in circularity was observed in 2 months group compared with the control (estimate = 0.050, 95% CI [0.014, 0.086], adjusted *p* = 0.046). No significant differences in circularity were detected for 1 day, 1 week, or 1 month.

Solidity also differed between groups. 1 day showed a significant increase in solidity compared with control (estimate = 0.056, 95% CI [0.021, 0.091], adjusted *p* = 0.004), while 2 months showed a larger increase (estimate = 0.076, 95% CI [0.043, 0.109], adjusted *p* < 0.001). Differences between 1 week and control and between 1 month and control were not statistically significant after adjustment.

### 3.3. Gene Expression Analysis

**Figure 8:**
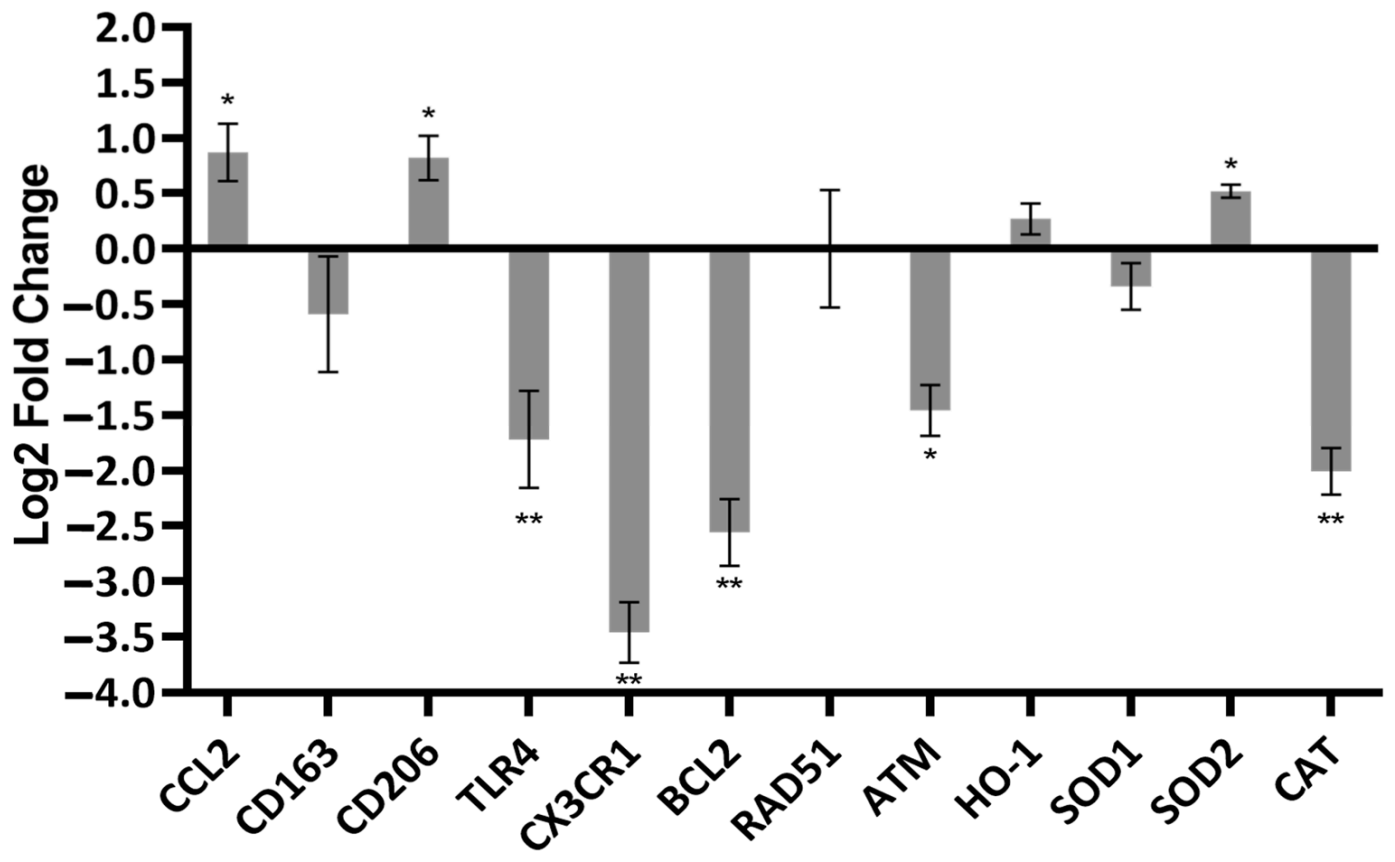
Analysis of gene expression in the mouse brain following total-body irradiation with 2 Gy of helium ions, assessed 8 weeks after exposure. Relative expression was normalized to the TBP reference gene and calculated using the 2^−ΔΔCt^ method. Data are presented as log2 fold change. Error bars represent standard deviation (SD) across biological replicates (n = 3). Between-group differences were assessed using the Mann–Whitney U test and corrected for multiple comparisons by use of the Benjamini–Hochberg Procedure (* q < 0.05, ** q < 0.01 versus the control group).

This study investigated the effects of helium ion irradiation on gene expression in the mouse brain using quantitative real-time PCR 8 weeks after exposure (Figure 8). The significant increase in CCL2 gene expression in brain tissue may reflect neuroimmune activation and a shift toward a pro-inflammatory-associated state, although CCL2 has also been associated with alternatively activated (M2-like) responses [38,39]. The CCL2 gene encodes a chemokine that plays a key role in recruiting monocytes and T-cells to sites of neuroinflammation, and its expression is typically enhanced in the acute phase following irradiation [40,41]. For instance, several studies have shown that CCL2 levels peak within the first hours after whole-body irradiation but normalize within 24 h post-exposure [42]. At higher doses, CCL2 can remain elevated for up to 30 days [43] or even six months [44], suggesting a transition to a chronic inflammatory state. Our data suggest that CCL2 is involved not only in the initiation but also in the maintenance of persistent inflammatory signaling at 8 weeks. Thus, the elevation of CCL2 at two months after irradiation observed in this study may reflect a sustained, low-grade neuroinflammatory state in the brain, potentially involving microglial activation and neurogenesis. Irradiated microglia can secrete CCL2 but barely express CCR2 [45,46]. Several studies have reported that high radiation doses (≥9 Gy) increase levels of CCR2+ macrophages and CCL2 in the mouse brain parenchyma [42,47,48,49]. In a mouse model of fractionated whole-brain irradiation with caesium-137, helium, or simulated space radiation, CSF1R blockade attenuated neuroinflammation and improved learning and memory [8,50,51]. The increased expression of CD206, a gene commonly associated with alternatively activated (M2) microglia, may indicate a shift toward tissue repair and attenuated inflammation [52,53]. Despite the increased expression of CD206 and CCL2, the expression of another M2-associated marker, CD163, showed a tendency to decrease, suggesting the presence of an atypical or mixed activation profile [54,55]. This pattern may reflect a mixed or transitional state of neuroimmune activation that does not strictly conform to the well-defined M1 (pro-inflammatory) or M2 (anti-inflammatory) subtypes [56]. Collectively, these findings are consistent with a partially adaptive neuroimmune response to ionizing radiation, in which features of alternative activation may coexist with signs of immune dysregulation; however, cellular or protein-level evidence would be required to define the microglial phenotype more precisely. Further support for altered neuroimmune signaling is provided by the decreased expression of TLR4 and CX3CR1. TLR4 is a pattern recognition receptor that activates the transcription of pro-inflammatory cytokines via the NF-κB pathway [57,58]. However, depending on the context, TLR4 can induce either a pro-inflammatory, pro-survival response associated with cell proliferation, or an anti-inflammatory response leading to cell death [59,60]. The CX3CR1 gene encodes a receptor predominantly expressed by microglia that mediates interaction with neuronal fractalkine and plays a crucial role in microglial function [61,62]. Its reduced expression may disrupt neuron-microglia communication [63]. Experimental models have shown that loss of CX3CR1 exacerbates neurodegeneration, particularly in the context of diseases such as Parkinson’s disease, amyotrophic lateral sclerosis, and diabetic retinopathy [64,65,66]. Thus, the suppression of CX3CR1 observed in this study may be associated with altered neuron-microglia communication and potentially enhanced neuronal vulnerability to radiation-induced neurotoxicity. Brain expression of redox-related genes was also altered post-irradiation, as evidenced by increased SOD2 expression coupled with decreased CAT expression. Redox homeostasis in the brain is also markedly altered post-irradiation, as evidenced by increased SOD2 expression coupled with decreased CAT expression. The SOD2 gene encodes mitochondrial superoxide dismutase, which converts superoxide into hydrogen peroxide and provides the first line of defense against oxidative stress in mitochondria [67,68]. Increased SOD2 expression may indicate altered mitochondrial antioxidant-response signaling following radiation exposure [69,70]. However, the decreased expression of the CAT gene, which is involved in neutralizing hydrogen peroxide, may suggest altered intracellular antioxidant-response capacity [71]. This imbalance can lead to hydrogen peroxide accumulation, promoting oxidative damage. Previous work has reported reduced activity of antioxidant enzymes upon exposure to electromagnetic and ionizing radiation, supporting the hypothesis of oxidative dysregulation [72]. Altered redox-related gene expression may be a factor in long-term mitochondrial dysfunction and neuronal damage, but functional or biochemical validation is required. Further evidence of cellular stress is provided by the decreased expression of ATM and BCL2 genes. ATM is a kinase activated by double-strand DNA breaks and is essential for DNA repair, cell cycle regulation, and maintaining genomic stability [73,74]. Reduced ATM expression suggests a potential weakening of the genome repair capacity, which could increase the risk of mutagenesis and delayed neuronal death [75]. BCL2 is an anti-apoptotic protein that maintains mitochondrial integrity and inhibits programmed cell death [76]. It is known that forced overexpression of BCL2 can delay radiation-induced apoptosis, underscoring its protective role [77]. The concurrent decrease in TLR4, ATM, and BCL2 expression may indicate a broad alteration in cellular defense and stress-response signaling, encompassing reduced inflammatory signaling, altered DNA-damage response, and a possibly lowered apoptotic threshold.

### 3.4. NMR Metabolomics Results

**Figure 9:**
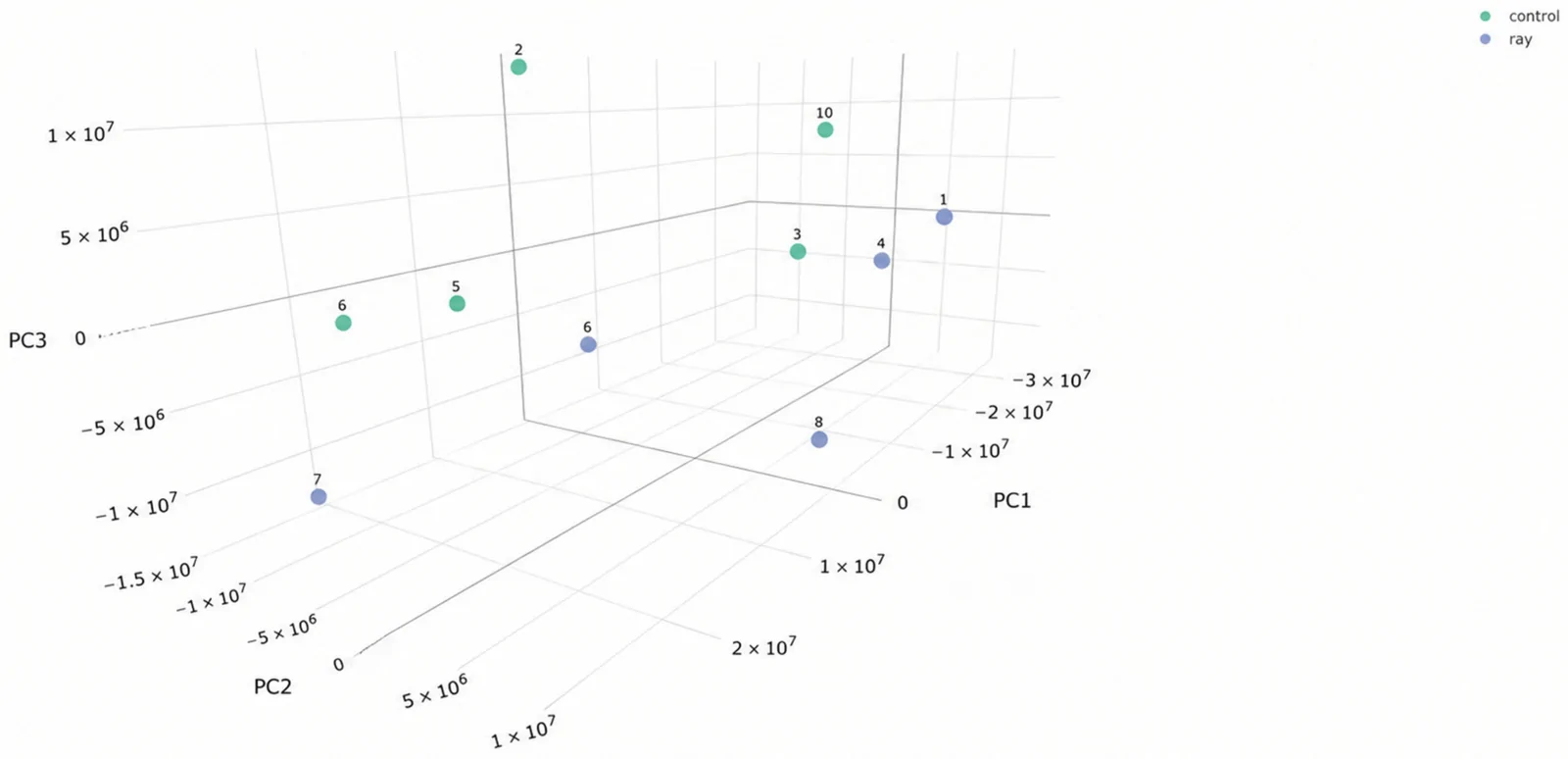
3D PCA score plot (PC1–PC2–PC3) of plasma ^1^H NMR spectra from control (green circles) and irradiated (blue circles) mice. PC1 = 53.7%, PC2 = 16.6%, PC3 = 18.3% of variance. The samples overlap substantially, and no clear separation between groups is observed.

NMR-based metabolomics allows the rapid, non-destructive analysis of low-molecular-weight metabolites in biological fluids. To assess metabolic alterations induced by helium ions, we compared the metabolite profiles in the blood plasma of control and irradiated mice using ^1^H NMR spectroscopy. To evaluate the efficacy of this method for detecting metabolic alterations induced by helium ions 8 weeks after exposure, we compared the relative metabolite profiles derived from group-averaged ^1^H NMR spectra in the blood plasma of mice from the control group (n = 5) and the group subjected to total-body irradiation at a dose of 2 Gy (n = 5). To assess metabolic alterations in the early delayed period after irradiation, plasma was collected from the study groups 8 weeks after irradiation, which corresponded to 1 week after the completion of behavioral testing. To determine whether there was a significant difference between the studied groups (control and irradiated), the obtained 1H-NMR spectra, which were first subjected to phase correction in the TOPSPIN program, were analyzed by multivariate statistical analysis using the principal component method in the metabom8 library [33]. Principal component analysis is the most popular unsupervised statistical method for processing data obtained by various omics techniques and does not require any prior assumptions or knowledge. Unsupervised methods usually represent the first step in data analysis, helping to visualize the data and detect possible outliers [78]. PCA was used as an exploratory visualization method. The 3D plot of values (PC1–PC2–PC3, Figure 9) shows that the control and irradiated samples partially overlap, with no clear separation observed between the groups. No formal multivariate testing (e.g., PERMANOVA) was performed.

**Figure 10:**
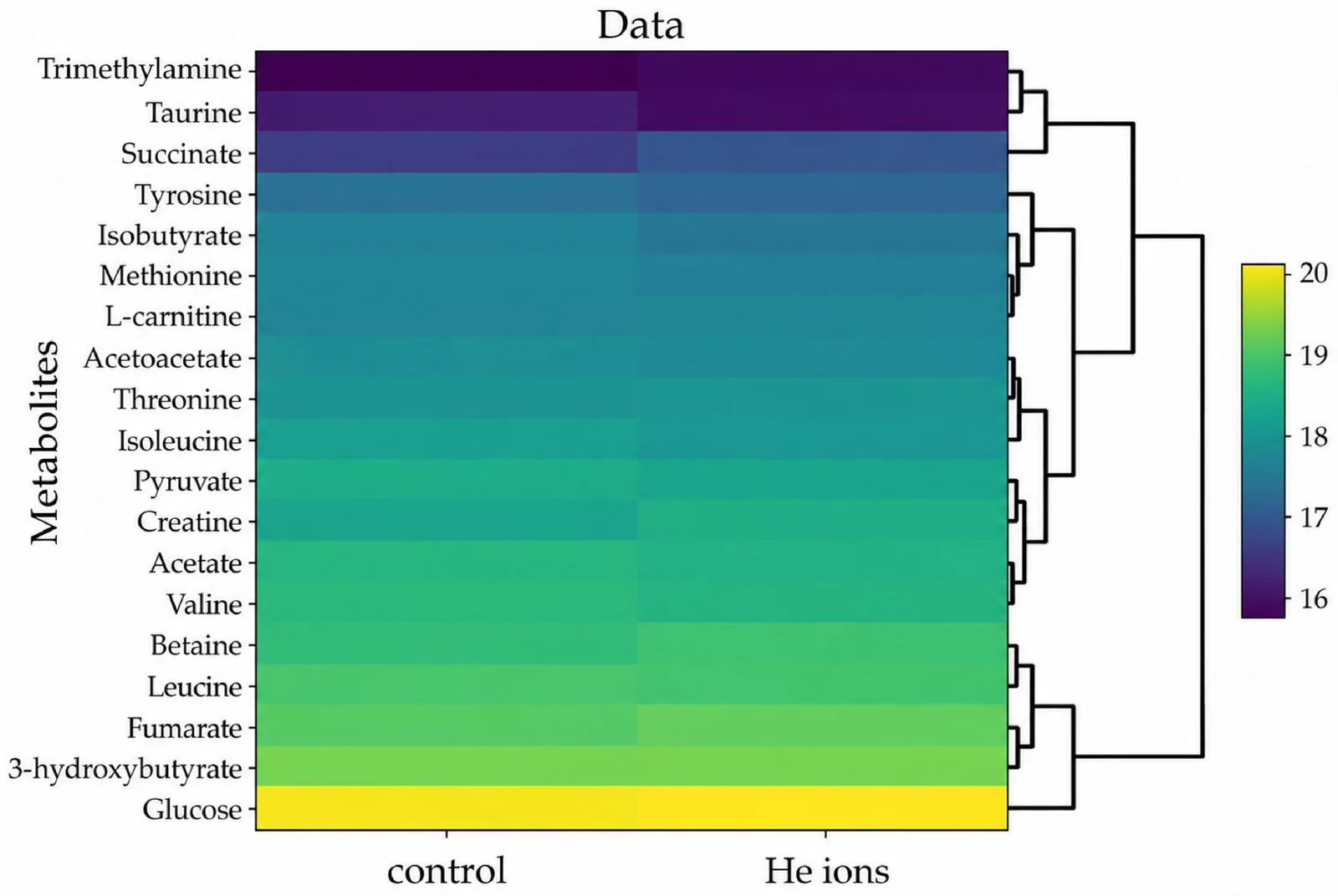
Heatmap of metabolic profiles of animals in the control and experimental groups.

To identify the difference in metabolites, mean NMR spectra for each study group were generated by summing the individually processed spectra within a group and then dividing the resultant summed spectrum by the number of animals in that group. A difference spectrum was calculated by subtracting the average spectrum of the irradiated group from the average spectrum of the control group (methodology adapted from [79]). The subtraction of the average spectra allowed for the precise identification of altered metabolites (19 metabolites). Signals corresponding to these metabolites in the average spectra were integrated (Supplementary Materials). Because this analysis was performed on group-averaged spectra, per-animal quantitative or semi-quantitative metabolite data were not available; therefore, no animal-level statistical analysis of metabolite concentrations was performed. The integrated signals from the control group were then compared to those from the irradiated group, and the differences were calculated as a percentage, representing the relative change in metabolite levels in the irradiated group compared to the control. These values are not absolute concentrations and should be interpreted as relative, exploratory trends. The heatmap of relative metabolite levels (Figure 10) shows a multidirectional pattern of changes in the irradiated group, with a tendency toward elevated succinate, creatine, betaine, trimethylamine, and fumarate, and reduced taurine, tyrosine, isobutyrate, and pyruvate. These changes should be regarded as trends rather than as statistically confirmed differences.

Because animals received whole-body irradiation, plasma metabolite changes cannot be attributed to the brain alone. The absence of brain-tissue metabolomic data and of tissue–plasma correlation analyses is a limitation of the present study.

## 4. Discussion

Given the significant interest in finding new, alternative, and more effective sources for cancer treatment, as well as global plans for deep space exploration, the task of assessing radiation risks for patients and astronauts, particularly the effects of helium ions on the CNS, has come to the forefront. Consequently, it is extremely important to assess the consequences of such radiation exposure on the central nervous system by evaluating changes in the microglial environment and its contribution to neuroinflammation, as well as to analyze changes in plasma metabolites and the expression of key DNA repair and redox potential genes at long-term time points after exposure [80].

In our study, we assessed the impact of the 2 Gy dose of whole-body helium ion irradiation on mouse behavior and memory. The 2 Gy dose used in this experiment substantially exceeds the anticipated contribution of helium ions during a long-duration space mission, and it corresponds to a conventional fractional dose used in tumor radiotherapy. However, it is delivered as a single acute whole-body exposure, which differs from both the chronic low-dose-rate GCR environment and the localized, treatment-planned delivery used in clinical helium-ion therapy. No statistically significant differences from the control group were detected in locomotor and exploratory activity or in hippocampus-dependent memory tests. These non-significant results are interpreted as an absence of statistically detectable group differences under the present experimental conditions (sample size, dose, time point, test sensitivity), rather than as evidence that irradiation had no behavioral effect. Both groups exhibited a reduction in escape latency across repeated Barnes-maze trials. Within this sample, no statistically detectable evidence was found that 2 Gy helium-ion irradiation altered the trajectory of escape latency during acquisition. However, the high proportion of latency values at the 180 s ceiling, together with the limited sample size, reduced the sensitivity of this analysis. Therefore, these findings do not establish equivalence between groups and cannot exclude small or moderate irradiation-related effects on spatial learning.

However, at the cellular level, the present analysis revealed a complex pattern of changes detectable at 8 weeks in the microglial population following irradiation. Importantly, when the hierarchical structure of the data was taken into account and the animal, rather than the individual cell, was considered the biological unit of analysis, the most consistent alteration was a reduction in cell density. Compared with controls, microglial cell density was significantly reduced in the 1 day, 1 month, and 2 months groups, whereas the difference in the 1 week group did not reach statistical significance. These findings indicate that the reduction in microglial abundance represents a more robust consequence of irradiation than changes in individual-cell morphology. The reduction in microglial cell number is consistent with previous evidence that ionizing radiation can affect microglial survival, proliferation, and differentiation [81]. A sustained decrease in the microglial population may have important implications for brain homeostasis because microglia contribute to immune surveillance, synaptic remodeling, phagocytosis, and maintenance of the neural microenvironment. Nevertheless, the present results should be interpreted as evidence of altered microglial abundance rather than direct evidence of impaired microglial viability, as the present experimental design does not distinguish between changes in cell survival, proliferation, recruitment, or phenotype-dependent detectability. In contrast to the reductions in Iba1-positive cell density, most morphological parameters showed limited and inconsistent differences between irradiated and control groups after accounting for repeated sampling of sections within individual animals and adjusting for multiple comparisons. No statistically significant group differences were detected for cell area, perimeter, aspect ratio, or roundness. The 8-week (2-month) group showed a relatively large reduction in cell area, but this difference did not meet the adjusted threshold for statistical significance. Selected morphological differences were nevertheless observed. Circularity was significantly increased at 2 months, and solidity was significantly increased at 1 day and 2 months. Increased solidity may be compatible with a more compact cell configuration and reduced complexity of the cell outline. However, these measures alone cannot establish a particular activation state or distinguish reactive, amoeboid, dystrophic, or other microglial phenotypes. Such conclusions require additional morphometric descriptors, molecular markers, and functional measurements.

Taken together, the findings indicate that whole-body irradiation was associated primarily with reduced Iba1-positive cell density in the sampled CA1 field, accompanied by selective rather than uniform morphological changes. Future studies incorporating time-specific control cohorts, microglia-enriched markers, and direct measurements of proliferation, cell death, and microglial function will be required to clarify the identity and biological significance of the observed changes.

In the literature, the majority of studies assessing the impact of helium ions on the cognitive abilities of laboratory animals have been conducted by modeling the unique multi-component galactic cosmic radiation field. For instance, a study by Krukowski et al. [13] demonstrated that exposure to this field (60% protons, 20% helium ions, and 20% oxygen ions) led to the development of anxiety, deficits in social interaction, and impaired recognition memory in male mice. These effects were accompanied by increased microgliosis in the hippocampus and synaptic losses 100 days after exposure. However, no cognitive or behavioral impairments were found in irradiated female mice, nor were there microgliosis or changes in synaptic content. In a study by Raber et al. [17], irradiated female mice exhibited increased anxiety, impaired recognition memory, and reduced memory retention in passive avoidance tests (observed in both males and females) two months after exposure. A non-statistically significant trend towards higher microglial activation (CD68+) was observed in the cerebral cortex of male mice irradiated at a dose of 0.5 Gy, while no such activation was present in female mice irradiated at doses of 0.25, 0.5, and 2 Gy. The authors found a positive correlation between microglial activation and brain-derived neurotrophic factor (BDNF) and attributed the differences in microglial activation between males and females to the varying percentage composition of ions in the radiation field used for the animals. In one of the more recent studies [82], multivariate principal component analysis also revealed sex-specific differences in the response of mice after whole-body irradiation at doses of 0.5 and 1 Gy. A study by [83] evaluated the behavioral and cognitive characteristics of C57BL/6J × DBA2/J F1 (B6D2F1) mice three months after irradiation with helium ions at doses of 0.21, 0.42, and 1.68 Gy. The authors found no changes in behavioral parameters in the open field test or depressive-like behavior in the forced swim test. However, cognitive impairments were observed in novel object recognition and passive avoidance tests, but not in tests for contextual or cued fear conditioning. In an earlier study assessing the late neuroinflammatory response, Krukowski et al. [8] found that irradiating mice with helium ions at doses of 0.15, 0.5, and 1 Gy did not affect recognition memory in the novel object recognition (NOR) test at early time points after irradiation (18+ days), while microglia were depleted. A deficit in this type of memory was detected 90+ days after exposure to doses of 0.15 Gy and 0.5 Gy. Analysis of the gene expression profile (96 chemokines and cytokines) revealed that acute microglial depletion alters the late neuroinflammatory response to cosmic radiation. For instance, levels of CCL2, CD206, CD163, and DUSP1 were reduced in the group receiving 0.5 Gy + PLX compared to the group receiving only 0.5 Gy. The authors found no changes in CCL2 and DUSP1 when comparing the 1 Gy and 1 Gy + PLX groups—two groups in which no behavioral impairments were observed. Interestingly, CD163 levels continued to decrease after PLX treatment, while CD206 increased with treatment. Another study on low doses of helium ions showed that 0.05 and 0.3 Gy increased the number of ED1+ activated microglia in the perirhinal cortex (PRC) [9], whereas 0.5 Gy had no effect on microglia. Levels of pro-inflammatory cytokines were elevated, and irradiated male mice demonstrated impaired functional connectivity between the hippocampal CA1 region and the PRC, which likely contributed to deficits in spatial, episodic, and recognition memory, impaired cognitive flexibility, and reduced fear extinction speed. The depressive-like behavior and anxiety effects observed in this study are not consistent with the previously mentioned research [8]. It is possible that microglia-mediated synaptic engulfment plays a role in the impaired functional connectivity and cognitive deficits induced by helium ions. Although increased microglial activation and cognitive deficits induced by helium ion irradiation may not be mediated by enhanced microglial synaptic engulfment, a concurrent reduction in its phagocytic activity could be a protective mechanism of the brain in response to radiation exposure. In a study by [10], the long-term adverse effect on memory from helium ion irradiation at doses of 0.15–0.5 Gy was investigated. It was demonstrated that transient microglial depletion after charged particle irradiation prevents any memory deficits when measured >90 days post-exposure. Furthermore, the recovery from memory deficits was found to be partially driven by at least three factors: minimized neuroinflammation, modulation of the repopulated microglial phenotype, and neuronal protein expression. Specifically, the reintroduced microglial population showed reduced expression of the phagocytic marker LAMP-1 and the complement mediator C5aR. The attenuation of cognitive deficits was associated with the upregulation of synapsin-1 and reduced levels of PSD-95 protein. These results clarified that the benefits of reducing microglia after irradiation may play a significant role in limiting memory deficits during space exploration. In the study by [51], mice were maintained on a PLX5622 diet to deplete microglia starting 2 weeks after whole-body irradiation with 0.3 Gy helium ions. At 4–6 weeks, it was found that irradiated animals on a regular diet exhibited a range of behavioral deficits involving the medial prefrontal cortex and hippocampus, along with increased microglial activation. In contrast, animals on the PLX5622 diet did not exhibit radiation-induced cognitive deficits, and the expression of both resting and activated microglia was almost completely suppressed, without affecting oligodendrocyte precursor cells throughout the brain. Additionally, the diet was found to attenuate the radiation-induced increase in postsynaptic density protein 95 (PSD-95) and preserve the density of mushroom-type spines, while other neuronal morphological features and intrinsic excitability electrophysiological measures were largely unaffected.

In our study, it was found that helium ion irradiation elicits a molecular response in the brain characterized by dysregulated immune activation and altered expression of redox-related genes. The significant changes in the expression of CCL2 and CD206 may suggest a shift toward an alternative or mixed activation profile of myeloid cells, potentially reflecting an incomplete or compensatory response. Simultaneously, the decreased expression of key genes responsible for pro-inflammatory signaling (TLR4), neuron-microglia communication (CX3CR1), antioxidant defense (CAT), DNA repair (ATM), and anti-apoptotic protection (BCL2) may reflect a state of altered molecular signaling in the brain at 8 weeks after irradiation. It is important to emphasize that the interpretation of these results must consider the specific experimental design. While previous studies have significantly contributed to our understanding of radiation-induced neuroinflammation and cognitive deficits, many of them involved total irradiation. It has been suggested that whole-body irradiation represents no significant limitation, as during space missions, particularly long-duration flights, exposure will be total-body, not limited to specific regions [8,13,20]. Furthermore, the doses applied in the literature often differ substantially from those used in the present study, limiting direct comparability. These differences in experimental design and radiation type highlight the necessity for developing models that more accurately reflect the nature of radiation exposure encountered during spaceflight.

It is known that the response of mice to high-dose radiation is characterized by a wide spectrum of metabolic alterations, including a slowdown in energy metabolism. In particular, elevated levels of taurine, which plays an important role in the development of the brain, retina, and kidneys, can serve as an early biomarker of high-dose radiation exposure [84]. The levels of most biomarkers identified via NMR decrease after radiation exposure, with the most significant reduction observed in metabolites involved in energy metabolism, namely succinate, citrate, and 2-oxoglutarate. Rats exposed to 3 Gy radiation exhibited a sharp decrease in citrate and 2-oxoglutarate levels [85]. In our investigation, NMR-based metabolomic analysis revealed a multidirectional pattern of changes in the plasma metabolic profile after helium ion irradiation, potentially reflecting the involvement of several interconnected metabolic pathways. Among the metabolites showing a tendency to increase, the most pronounced elevation was observed for succinate, creatine, betaine, trimethylamine, and fumarate. A more moderate increase was observed for threonine and glucose. The parallel tendency toward elevated succinate and fumarate may be consistent with alterations in intermediate mitochondrial metabolism, although flux measurements would be required to confirm this. The reduction in taurine may reflect changes in sulfur-containing amino acid metabolism and antioxidant homeostasis, but should not be interpreted as a direct marker of oxidative stress. The tendency toward reduced BCAAs (valine, isoleucine, leucine) may indicate alterations in BCAA metabolism, although flux measurements would be required to confirm this. Because irradiation was delivered to the whole body, plasma metabolites may originate from multiple irradiated tissues and cannot be attributed specifically to the brain. The observed systemic metabolic changes may therefore reflect a coordinated multi-tissue response to irradiation rather than a brain-specific effect. Brain-tissue metabolomics and tissue–plasma correlation analyses would be required to determine whether the plasma profile reflects CNS-related metabolic alterations. Given the small sample size (n = 5 per group), the exploratory nature of the analysis, and the lack of correction for multiple comparisons, the individual metabolite changes should be considered as trends rather than as statistically confirmed differences. In addition, PCA was used as an exploratory visualization method only; because the confidence ellipses of the control and irradiated groups overlapped substantially, and because formal multivariate testing (e.g., PERMANOVA) was not performed, the absence of a clear group separation should be interpreted as a descriptive observation rather than as a formal statistical result. All plasma samples (5 control and 5 irradiated) were included in the analysis; no samples were excluded. The follow-up period in this study was limited to 8 weeks. Although the observed molecular and cellular alterations persisted across the sampled time points, they were not accompanied by overt behavioral deficits. These changes should therefore be described as early delayed alterations rather than established long-term pathology. Their progression at later time points remains to be determined.

## 5. Conclusions

Exposure to 2 Gy of helium ions did not produce overt behavioral or cognitive deficits at 8 weeks after irradiation. However, molecular, cellular, and systemic metabolic alterations were detectable at this time point, including reduced microglial density, changes in microglial morphology, altered expression of neuroimmune, redox-related, and DNA-repair genes, and a distinct plasma metabolomic profile. Because these changes were observed in the absence of major behavioral deficits, we hypothesize that these early molecular and cellular changes may predispose to functional deficits at later time points or under combined stressors, but this remains to be tested in longer-term studies. They warrant longer-term investigation, including behavioral testing at later time points and, ideally, brain-tissue metabolomics, to determine whether they represent adaptive responses, subclinical alterations, or precursors of future dysfunction. Our findings underscore the value of multimodal assessment beyond behavioral testing for evaluating the effects of charged-particle exposure, and they highlight the importance of extended follow-up in future studies.

## 6. Limitations

Several limitations of this study should be acknowledged.

First, only male mice were used. Sex differences in radiation-induced neuroinflammation, microglial responses, and cognitive outcomes have been reported in the literature, and our findings may therefore not be generalizable to female animals.

Second, the study used a single relatively high dose of 2 Gy helium ions delivered as an acute whole-body exposure. This does not reflect the chronic, low-dose-rate, mixed-field galactic cosmic ray environment encountered during deep-space missions, nor does it reflect the localized, treatment-planned delivery used in clinical helium-ion therapy. The 2 Gy dose also substantially exceeds the anticipated contribution of helium ions during a long-duration space mission. The present results should therefore be interpreted as fundamental radiobiological observations rather than direct predictions of spaceflight or radiotherapy outcomes.

Third, oxidative damage was not directly measured. Although we observed changes in the expression of redox-related genes (SOD2, CAT), these are indirect indicators and do not establish oxidative stress as a mechanism.

Fourth, microglia-specific molecular analyses were not performed. The gene expression analysis was carried out on whole-brain tissue, and the observed changes therefore cannot be attributed specifically to microglia. Likewise, microglial function was not assessed, and the changes in microglial density and morphology cannot be interpreted as evidence of microglial dysfunction.

Fifth, the metabolomic measurements were performed on blood plasma. Because irradiation was delivered to the whole body, plasma metabolites may originate from multiple irradiated tissues and cannot be attributed specifically to the brain. Brain-tissue metabolomics and tissue–plasma correlation analyses would be required to determine whether the plasma profile reflects CNS-related metabolic alterations.

Sixth, the sample sizes were limited: n = 10 per group for behavioral testing, n = 3 per group for qPCR, n = 5 per group for NMR metabolomics, and n = 3–5 per group for microglial immunohistochemistry. In particular, the small sample size for the metabolomic analysis (n = 5 per group), together with the lack of correction for multiple comparisons and the absence of formal multivariate testing, means that the metabolite changes should be considered exploratory and hypothesis-generating. The use of a pooled control group for the microglial analysis is a further limitation, as it does not fully exclude time-dependent variability in control microglial parameters.

Finally, the follow-up period was limited to 8 weeks. Although molecular and cellular alterations were detectable at this time point and were not accompanied by overt behavioral deficits, longer-term functional follow-up would be required to determine whether these early changes progress, resolve, or have functional consequences at later time points or under combined stressors.

## Figures and Tables

**Table 1:** Gene-specific primers for qPCR used in the experiment.

Gene Name	Primer Sequence
CCL2	F: CTGCTGTTCACAGTTGCCG
	R: GCACAGACCTCTCTCTTGAGC
CD163	F: AAGATGCTGGAGTGACCTGC
	R: TCTTCCTAAGCATCGGTGGC
CD206	F: ATGGATTGCCCTGAACAGCA
	R: TGTACCGCACCCTCCATCTA
TLR4	F: AGATCTGAGCTTCAACCCCTTG
	R: AGAGGTGGTGTAAGCCATGC
CX3CR1	F: TGCAGAAGTTCCCTTCCCATC
	R: GCCCAAATAACAGGCCTCAG
BCL2	F: AACATCGCCCTGTGGATGAC
	R: AAACAGAGGTCGCATGCTGG
RAD51	F: AGCCGGTTACCATACAGTGG
	R: AAACTCAGTTGCCGTGGTGA
ATM	F: AACCATGCTTGCTGTTGTCG
	R: AATCCAGCCAGAAAGCGTCA
HO-1	F: CAGAAGAGGCTAAGACCGCC
	R: ATCTTGCACCAGGCTAGCAG
SOD1	F: GGAAGCATGGCGATGAAAGC
	R: CCCATGCTGGCCTTCAGTTA
SOD2	F: CCACCGAGGAGAAGTACCAC
	R: GGGTTCTCCACCACCCTTAG
CAT	F: GCGGATTCCTGAGAGAGTGG
	R: TGTGGAGAATCGAACGGCAA
TBP	F: ACAGCCTTCCACCTTATGCTC
	R: CTGTGGAGTAAGTCCTGTGCC

## Data Availability

The original contributions presented in this study are included in the article/Supplementary Material. Further inquiries can be directed to the corresponding author(s).

## Supplementary Materials

The following supporting information can be downloaded at: https://www.mdpi.com/article/10.3390/biology15201819/s1, Figure S1: 1H NMR spectra obtained for the plasma of each animal; Figure S2: 2D PCA score plot (PC1–PC2–PC3) of plasma 1H NMR spectra; Figure S3: Mean 1H NMR spectra for the experimental groups.

## References

[1] Levy R., Fabrikant J., Frankel K., Phillips M., Lyman J., Lawrence J., Tobias C. (1991). Heavy-Charged-Particle Radiosurgery of the Pituitary Gland: Clinical Results of 840 Patients. Stereotact. Funct. Neurosurg..

[2] Mishra K., Quivey J., Daftari I., Weinberg V., Cole T., Patel K., Castro J., Phillips T., Char D. (2015). Long-Term Results of the UCSF-LBNL Randomized Trial: Charged Particle with Helium Ion Versus Iodine-125 Plaque Therapy for Choroidal and Ciliary Body Melanoma. Int. J. Radiat. Oncol. Biol. Phys..

[3] Krämer M., Scifoni E., Schuy C., Rovituso M., Tinganelli W., Maier A., Kaderka R., Kraft-Weyrather W., Brons S., Tessonnier T. (2016). Helium Ions for Radiotherapy? Physical and Biological Verifications of a Novel Treatment Modality. Med. Phys..

[4] Tessonnier T., Mairani A., Chen W., Sala P., Cerutti F., Ferrari A., Haberer T., Debus J., Parodi K. (2018). Proton and Helium Ion Radiotherapy for Meningioma Tumors: A Monte Carlo-Based Treatment Planning Comparison. Radiat. Oncol..

[5] Cucinotta F. (2014). Space Radiation Risks for Astronauts on Multiple International Space Station Missions. PLoS ONE.

[6] De Wet W., Townsend L. (2017). A Calculation of the Radiation Environment on the Martian Surface. Life Sci. Space Res..

[7] Nelson G. (2016). Space Radiation and Human Exposures, a Primer. Radiat. Res..

[8] Krukowski K., Feng X., Paladini M., Chou A., Sacramento K., Grue K., Riparip L.-K., Jones T., Campbell-Beachler M., Nelson G. (2018). Temporary Microglia-Depletion After Cosmic Radiation Modifies Phagocytic Activity and Prevents Cognitive Deficits. Sci. Rep..

[9] Parihar V., Maroso M., Syage A., Allen B., Angulo M., Soltesz I., Limoli C. (2018). Persistent Nature of Alterations in Cognition and Neuronal Circuit Excitability After Exposure to Simulated Cosmic Radiation in Mice. Exp. Neurol..

[10] Rosi S. (2018). The Final Frontier: Transient Microglia Reduction After Cosmic Radiation Exposure Mitigates Cognitive Impairments and Modulates Phagocytic Activity. Brain Circ..

[11] Hassler D., Zeitlin C., Wimmer-Schweingruber R., Ehresmann B., Rafkin S., Eigenbrode J., Brinza D., Weigle G., Böttcher S., Böhm E. (2014). Mars’ Surface Radiation Environment Measured with the Mars Science Laboratory’s Curiosity Rover. Science.

[12] Zeitlin C., Hassler D., Cucinotta F., Ehresmann B., Wimmer-Schweingruber R., Brinza D., Kang S., Weigle G., Bottcher S., Bohm E. (2013). Measurements of Energetic Particle Radiation in Transit to Mars on the Mars Science Laboratory. Science.

[13] Krukowski K., Grue K., Frias E., Pietrykowski J., Jones T., Nelson G., Rosi S. (2018). Female Mice Are Protected from Space Radiation-Induced Maladaptive Responses. Brain Behav. Immun..

[14] Poulose S., Bielinski D., Carrihill-Knoll K., Rabin B., Shukitt-Hale B. (2011). Exposure to 16O-Particle Radiation Causes Aging-Like Decrements in Rats Through Increased Oxidative Stress, Inflammation and Loss of Autophagy. Radiat. Res..

[15] Raber J., Allen A., Sharma S., Allen B., Rosi S., Olsen R., Davis M., Eiwaz M., Fike J., Nelson G. (2015). Effects of Proton and Combined Proton and ^56^Fe Radiation on the Hippocampus. Radiat. Res..

[16] Raber J., Yamazaki J., Torres E., Kirchoff N., Stagaman K., Sharpton T., Turker M., Kronenberg A. (2019). Combined Effects of Three High-Energy Charged Particle Beams Important for Space Flight on Brain, Behavioral and Cognitive Endpoints in B6D2F1 Female and Male Mice. Front. Physiol..

[17] Raber J., Fuentes Anaya A., Torres E., Lee J., Boutros S., Grygoryev D., Hammer A., Kasschau K., Sharpton T., Turker M. (2020). Effects of Six Sequential Charged Particle Beams on Behavioral and Cognitive Performance in B6D2F1 Female and Male Mice. Front. Physiol..

[18] Rola R., Otsuka S., Obenaus A., Nelson G., Limoli C., VandenBerg S., Fike J. (2004). Indicators of Hippocampal Neurogenesis Are Altered by 56Fe-Particle Irradiation in a Dose-Dependent Manner. Radiat. Res..

[19] Shukitt-Hale B., Casadesus G., McEwen J.J., Rabin B.M., Joseph J.A. (2000). Spatial Learning and Memory Deficits Induced by ^56^Fe Radiation Exposure. Radiat. Res..

[20] Rabin B., Shukitt-Hale B., Carrihill-Knoll K., Gomes S. (2014). Comparison of the Effects of Partial- or Whole-Body Exposures to ^16^O Particles on Cognitive Performance in Rats. Radiat. Res..

[21] Wang Y., Bao X., Zhang Y., Wu Q. (2024). The Current Research Status of the Mechanisms and Treatment of Radioactive Brain Injury. Am. J. Cancer Res..

[22] Inaniwa T., Suzuki M., Hyun Lee S., Mizushima K., Iwata Y., Kanematsu N., Shirai T. (2020). Experimental Validation of Stochastic Microdosimetric Kinetic Model for Multi-Ion Therapy Treatment Planning with Helium-, Carbon-, Oxygen-, and Neon-Ion Beams. Phys. Med. Biol..

[23] Tessonnier T., Ecker S., Besuglow J., Naumann J., Mein S., Longarino F., Ellerbrock M., Ackermann B., Winter M., Brons S. (2023). Commissioning of Helium Ion Therapy and the First Patient Treatment with Active Beam Delivery. Int. J. Radiat. Oncol. Biol. Phys..

[24] Knäusl B., Fuchs H., Dieckmann K., Georg D. (2016). Can Particle Beam Therapy Be Improved Using Helium Ions?—A Planning Study Focusing on Pediatric Patients. Acta Oncol..

[25] Tessonnier T., Mairani A., Brons S., Sala P., Cerutti F., Ferrari A., Haberer T., Debus J., Parodi K. (2017). Helium Ions at the Heidelberg Ion Beam Therapy Center: Comparisons Between FLUKA Monte Carlo Code Predictions and Dosimetric Measurements. Phys. Med. Biol..

[26] Mairani A., Mein S., Blakely E., Debus J., Durante M., Ferrari A., Fuchs H., Georg D., Grosshans D., Guan F. (2022). Roadmap: Helium Ion Therapy. Phys. Med. Biol..

[27] Casciati A., Dobos K., Antonelli F., Benedek A., Kempf S.J., Bellés M., Balogh A., Tanori M., Heredia L., Atkinson M.J. (2016). Age-related effects of X-ray irradiation on mouse hippocampus. Oncotarget.

[28] Kempf S.J., Casciati A., Buratovic S., Janik D., von Toerne C., Ueffing M., Neff F., Moertl S., Stenerlöw B., Saran A. (2014). The cognitive defects of neonatally irradiated mice are accompanied by changed synaptic plasticity, adult neurogenesis and neuroinflammation. Mol. Neurodegener..

[29] Buratovic S., Stenerlöw B., Fredriksson A., Sundell-Bergman S., Viberg H., Eriksson P. (2014). Neonatal exposure to a moderate dose of ionizing radiation causes behavioural defects and altered levels of tau protein in mice. Neurotoxicology.

[30] Mouton L., Etienne O., Feat-Vetel J., Barrière D.A., Pérès E.A., Boumezbeur F., Boussin F.D., Le Bihan D. (2021). Noninvasive Assessment of Neurodevelopmental Disorders after In Utero Irradiation in Mice: An In Vivo Anatomical and Diffusion MRI Study. Radiat. Res..

[31] Liu Q., Huang Y., Duan M., Yang Q., Ren B., Tang F. (2022). Microglia as Therapeutic Target for Radiation-Induced Brain Injury. Int. J. Mol. Sci..

[32] Ung M.C., Garrett L., Dalke C., Leitner V., Dragosa D., Hladik D., Neff F., Wagner F., Zitzelsberger H., Miller G. (2021). Dose-dependent long-term effects of a single radiation event on behaviour and glial cells. Int. J. Radiat. Biol..

[33] Kimhofer T. Metabom8 (Version 1.0.0), 2021. https://github.com/tkimhofer/metabom8.

[34] Dieterle F., Ross A., Schlotterbeck G., Senn H. (2006). Probabilistic quotient normalization as robust method to account for dilution of complex biological mixtures. Application in 1 H NMR metabonomics. Anal. Chem..

[35] Lalonde R., Strazielle C. (2008). Relations Between Open-Field, Elevated Plus-Maze, and Emergence Tests as Displayed by C57/BL6J and BALB/c Mice. J. Neurosci. Methods.

[36] van Gaalen M.M., Steckler T. (2000). Behavioural analysis of four mouse strains in an anxiety test battery. Behav. Brain Res..

[37] Kovalev G.I., Kondrakhin E.A., Salimov R.M. (2013). Behavioral and Neuroreceptor Differences in C57BL/6 and BALB/c Mice. Neurochem. J..

[38] Selenica M., Alvarez J., Nash K., Lee D., Cao C., Lin X., Reid P., Mouton P., Morgan D., Gordon M. (2013). Diverse Activation of Microglia by Chemokine (C-C Motif) Ligand 2 Overexpression in Brain. J. Neuroinflammation.

[39] Zhang C., Zhou Y., Gao Y., Zhu Z., Zeng Q., Yan W., Yuan A., Hu J., Fang Y. (2022). Radiated Glioblastoma Cell-Derived Exosomal circ_0012381 Induce M2 Polarization of Microglia to Promote the Growth of Glioblastoma by CCL2/CCR2 Axis. J. Transl. Med..

[40] Ge S., Murugesan N., Pachter J. (2009). Astrocyte- and Endothelial-Targeted CCL2 Conditional Knockout Mice: Critical Tools for Studying the Pathogenesis of Neuroinflammation. J. Mol. Neurosci..

[41] Qian B., Li J., Zhang H., Kitamura T., Zhang J., Campion L., Kaiser E., Snyder L., Pollard J. (2011). CCL2 Recruits Inflammatory Monocytes to Facilitate Breast-Tumour Metastasis. Nature.

[42] Lee S., Haditsch U., Cord B., Guzman R., Kim S., Boettcher C., Priller J., Ormerod B., Palmer T. (2013). Absence of CCL2 Is Sufficient to Restore Hippocampal Neurogenesis Following Cranial Irradiation. Brain Behav. Immun..

[43] Han W., Umekawa T., Zhou K., Zhang X., Ohshima M., Dominguez C., Harris R., Zhu C., Blomgren K. (2016). Cranial Irradiation Induces Transient Microglia Accumulation, Followed by Long-Lasting Inflammation and Loss of Microglia. Oncotarget.

[44] Moravan M., Olschowka J., Williams J., O’Banion M. (2011). Cranial Irradiation Leads to Acute and Persistent Neuroinflammation with Delayed Increases in T-Cell Infiltration and CD11c Expression in C57BL/6 Mouse Brain. Radiat. Res..

[45] Osman A., Sun Y., Burns T., He L., Kee N., Oliva-Vilarnau N., Alevyzaki A., Zhou K., Louhivuori L., Uhlen P. (2020). Radiation Triggers a Dynamic Sequence of Transient Microglial Alterations in Juvenile Brain. Cell Rep..

[46] Whitelaw B., Tanny S., Johnston C., Majewska A., O’Banion M., Marples B. (2021). In Vivo Imaging of the Microglial Landscape After Whole Brain Radiation Therapy. Int. J. Radiat. Oncol. Biol. Phys..

[47] Gan C., Li W., Xu J., Pang L., Tang L., Yu S., Li A., Ge H., Huang R., Cheng H. (2023). Advances in the study of the molecular biological mechanisms of radiation-induced brain injury. Am. J. Cancer Res..

[48] Morganti J., Jopson T., Liu S., Gupta N., Rosi S. (2014). Cranial Irradiation Alters the Brain’s Microenvironment and Permits CCR2+ Macrophage Infiltration. PLoS ONE.

[49] Markarian M., Krattli R., Baddour J., Alikhani L., Giedzinski E., Usmani M., Agrawal A., Baulch J., Tenner A., Acharya M. (2021). Glia-Selective Deletion of Complement C1q Prevents Radiation-Induced Cognitive Deficits and Neuroinflammation. Cancer Res..

[50] Feng X., Jopson T., Paladini M., Liu S., West B., Gupta N., Rosi S. (2016). Colony-Stimulating Factor 1 Receptor Blockade Prevents Fractionated Whole-Brain Irradiation-Induced Memory Deficits. J. Neuroinflammation.

[51] Allen B., Syage A., Maroso M., Baddour A., Luong V., Minasyan H., Giedzinski E., West B., Soltesz I., Limoli C. (2020). Mitigation of Helium Irradiation-Induced Brain Injury by Microglia Depletion. J. Neuroinflammation.

[52] Bai X., Hao L., Guo Y., Shi X., Wu W. (2021). Bone Marrow Stromal Cells Reverse the Microglia Type from Pro-Inflammatory Tumour Necrosis Factor Microglia to Anti-Inflammatory CD206 Microglia of Middle Cerebral Artery Occlusion Rats through Triggering Secretion of CX3CL1. Folia Neuropathol..

[53] Deng S., Jin P., Liu S., He Y., Sherchan P., Zhang J., Gong Y., Tang J. (2023). Recruitment of Regulatory T Cells with rCCL17 Promotes M2 Microglia/Macrophage Polarization Through TGFβ/TGFβR/Smad2/3 Pathway in a Mouse Model of Intracerebral Hemorrhage. Exp. Neurol..

[54] Wedekind H., Walz K., Buchbender M., Kitz J., Grabenbauer G., Fietkau R., Hecht M., Distel L. (2022). Head and Neck Tumor Cells Treated with Hypofractionated Irradiation Die via Apoptosis and Are Better Taken Up by M1-Like Macrophages. Strahlenther. Onkol..

[55] Chen S., Ni S., Liu C., He M., Pan Y., Cui P., Wang C., Ni X. (2023). Neglected Immunoregulation: M2 Polarization of Macrophages Triggered by Low-Dose Irradiation Plays an Important Role in Bone Regeneration. J. Cell Mol. Med..

[56] Italiani P., Boraschi D. (2014). From Monocytes to M1/M2 Macrophages: Phenotypical vs. Functional Differentiation. Front. Immunol..

[57] Teng W., Wang L., Xue W., Guan C. (2009). Activation of TLR4-Mediated NFkappaB Signaling in Hemorrhagic Brain in Rats. Mediat. Inflamm..

[58] Moretti I., Lerario A., Trombetta-Lima M., Weller M., Le Rhun E., Oba-Shinjo S., Marie S., Silber C. (2021). Late p65 Nuclear Translocation in Glioblastoma Cells Indicates Non-Canonical TLR4 Signaling and Activation of DNA Repair Genes. Sci. Rep..

[59] Cribbs D., Berchtold N., Perreau V., Coleman P., Rogers J., Tenner A., Cotman C. (2012). Extensive Innate Immune Gene Activation Accompanies Brain Aging, Increasing Vulnerability to Cognitive Decline and Neurodegeneration: A Microarray Study. J. Neuroinflammation.

[60] Rahimifard M., Maqbool F., Moeini-Nodeh S., Niaz K., Abdollahi M., Braidy N., Nabavi S., Nabavi S. (2017). Targeting the TLR4 Signaling Pathway by Polyphenols: A Novel Therapeutic Strategy for Neuroinflammation. Ageing Res. Rev..

[61] Dénes Á., Ferenczi S., Halász J., Környei Z., Kovács K. (2008). Role of CX3CR1 (Fractalkine Receptor) in Brain Damage and Inflammation Induced by Focal Cerebral Ischemia in Mouse. J. Cereb. Blood Flow. Metab..

[62] Clark A., Staniland A., Malcangio M. (2011). Fractalkine/CX3CR1 Signalling in Chronic Pain and Inflammation. Curr. Pharm. Biotechnol..

[63] Prinz M., Priller J. (2010). Tickets to the Brain: Role of CCR2 and CX3CR1 in Myeloid Cell Entry in the CNS. J. Neuroimmunol..

[64] Lv B., Huo F., Zhu Z., Xu Z., Liu T., Ma X., Zhang X. (2016). Crocin Upregulates CX3CR1 Expression by Suppressing NF-κB/YY1 Signaling and Inhibiting Lipopolysaccharide-Induced Microglial Activation. Neurochem. Res..

[65] Wolf Y., Yona S., Kim K., Jung S. (2013). Microglia, Seen from the CX3CR1 Angle. Front. Cell Neurosci..

[66] Castro-Sánchez S., García-Yagüe Á.J., López-Royo T., Casarejos M., Lanciego J.L., Lastres-Becker I. (2018). Cx3cr1-Deficiency Exacerbates Alpha-Synuclein-A53T Induced Neuroinflammation and Neurodegeneration in a Mouse Model of Parkinson’s Disease. Glia.

[67] Flynn J., Melov S. (2013). SOD2 in Mitochondrial Dysfunction and Neurodegeneration. Free Radic. Biol. Med..

[68] Kim Y., Gupta Vallur P., Phaëton R., Mythreye K., Hempel N. (2017). Insights into the Dichotomous Regulation of SOD2 in Cancer. Antioxidants.

[69] Jung C., Kim E., Song J., Park J., Hong S. (2019). Mitochondrial Superoxide Dismutase 2 Mediates γ-Irradiation-Induced Cancer Cell Invasion. Exp. Mol. Med..

[70] Hosoki A., Yonekura S., Zhao Q., Wei Z., Takasaki I., Tabuchi Y., Wang L., Hasuike S., Nomura T., Tachibana A. (2012). Mitochondria-Targeted Superoxide Dismutase (SOD2) Regulates Radiation Resistance and Radiation Stress Response in HeLa Cells. J. Radiat. Res..

[71] Ighodaro O., Akinloye O. (2018). First Line Defence Antioxidants-Superoxide Dismutase (SOD), Catalase (CAT) and Glutathione Peroxidase (GPX): Their Fundamental Role in the Entire Antioxidant Defence Grid. Alex. J. Med..

[72] Varghese R., Majumdar A., Kumar G., Shukla A. (2018). Rats Exposed to 2.45 GHz of Non-Ionizing Radiation Exhibit Behavioral Changes with Increased Brain Expression of Apoptotic Caspase 3. Pathophysiology.

[73] Lavin M., Kozlov S. (2007). ATM Activation and DNA Damage Response. Cell Cycle.

[74] Heinloth A., Shackelford R., Innes C., Bennett L., Li L., Amin R., Sieber S., Flores K., Bushel P., Paules R. (2003). ATM-Dependent and -Independent Gene Expression Changes in Response to Oxidative Stress, Gamma Irradiation, and UV Irradiation. Radiat. Res..

[75] Carlessi L., De Filippis L., Lecis D., Vescovi A., Delia D. (2009). DNA-Damage Response, Survival and Differentiation in Vitro of a Human Neural Stem Cell Line in Relation to ATM Expression. Cell Death Differ..

[76] Siddiqui W., Ahad A., Ahsan H. (2015). The Mystery of BCL2 Family: Bcl-2 Proteins and Apoptosis: An Update. Arch. Toxicol..

[77] Itamochi H., Yamasaki F., Sudo T., Kigawa J., Terakawa N. (2006). Reduction of Radiation-Induced Apoptosis by Specific Expression of Bcl-2 in Normal Cells. Cancer Gene Ther..

[78] Trygg J., Holmes E., Lundstedt T. (2007). Chemometrics in metabonomics. J. Proteome Res..

[79] Larkin J.R., Anthony S., Johanssen V.A., Yeo T., Sealey M., Yates A.G., Smith C.F., Claridge T.D., Nicholson B.D., Moreland J.-A. (2022). Metabolomic Biomarkers in Blood Samples Identify Cancers in a Mixed Population of Patients with Nonspecific Symptoms. Clin. Cancer Res..

[80] Miry O., Zhang X., Vose L., Giedzinski E., Fike J., Limoli C., Nelson G., Raber J. (2021). Life-Long Brain Compensatory Responses to Galactic Cosmic Radiation Exposure. Sci. Rep..

[81] Pariset E., Malkani S., Cekanaviciute E., Costes S. (2020). Ionizing Radiation-Induced Risks to the Central Nervous System and Countermeasures in Cellular and Rodent Models. Int. J. Radiat. Biol..

[82] Krukowski K., Grue K., Becker M., Elizarraras E., Frias E., Halvorsen A., Koenig-Zanoff M., Frattini V., Nimmagadda H., Feng X. (2021). The Impact of Deep Space Radiation on Cognitive Performance: From Biological Sex to Biomarkers to Countermeasures. Sci. Adv..

[83] Raber J. (2018). Effects of Space Radiation on the Brain and Behavior. Int. J. Mol. Sci..

[84] Chen C., Brenner D.J., Brown T.R. (2011). Identification of Urinary Biomarkers from X-Irradiated Mice Using NMR Spectroscopy. Radiat. Res..

[85] Lanz C., Patterson A., Slavík J., Krausz K., Ledermann M., Gonzalez F., Idle J. (2009). Radiation Metabolomics. Biomarker Discovery in the Urine of Gamma-Irradiated Rats Using a Simplified Metabolomics Protocol of Gas Chromatography-Mass Spectrometry Combined with Random Forests Machine Learning Algorithm. Radiat. Res..

