# Supplementary material for: Impact of Helium Ions on Mouse Behavior, Memory, Microglia, and Blood Metabolome
Source: Biology (Basel). 2026 Oct 12;15(20):1819. doi: 10.3390/biology15201819 (PMC13701629; doi:10.3390/biology15201819)
Supplement: Supplementary file 1 [file biology-15-01819-s001.zip › biology-4583226-supplementary.pdf]

NMR_Supplementary

1H NMR spectra were obtained for the plasma of each animal (Figure S1):

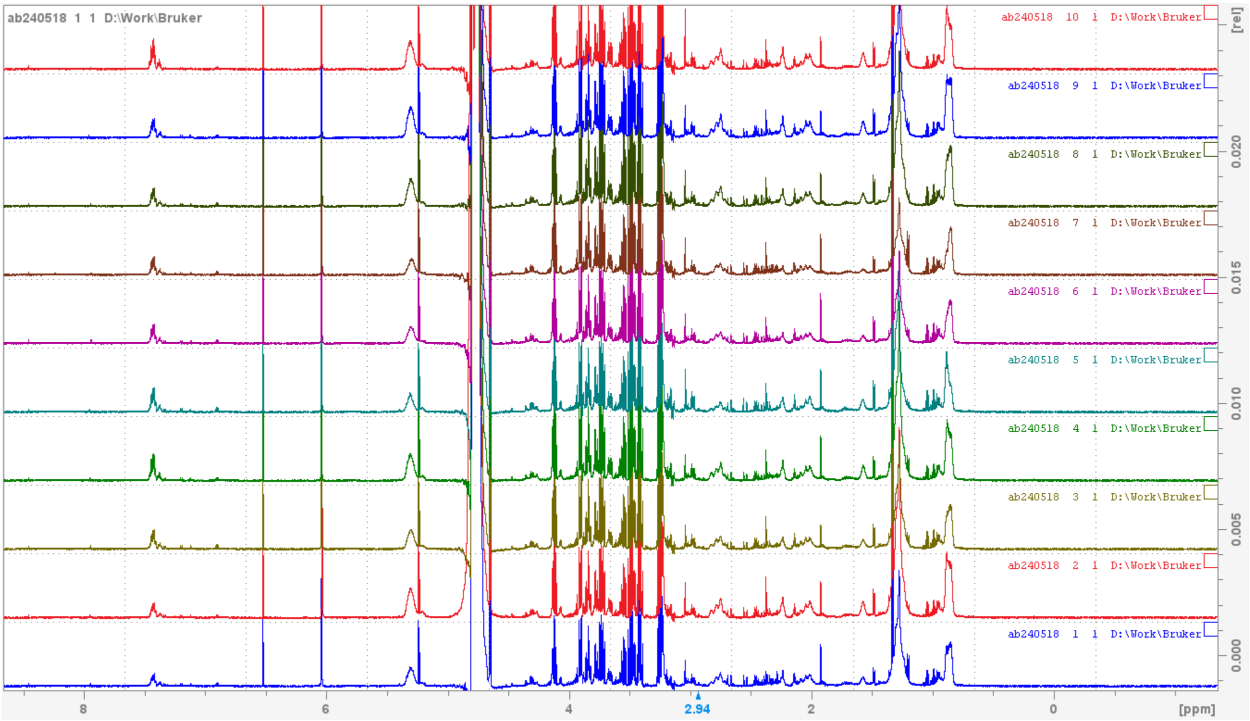


Multivariate statistical analysis (PCA) was used to assess differences between groups resulting from changes in metabolite concentrations. PCA is the most popular unsupervised statistical method for processing data obtained via various omics technologies and requires no prior assumptions or knowledge.

Figure S2: 2D PCA score plot (PC1–PC2–PC3) of plasma 1H NMR spectra from control (red circles) and irradiated (blue triangles) mice.

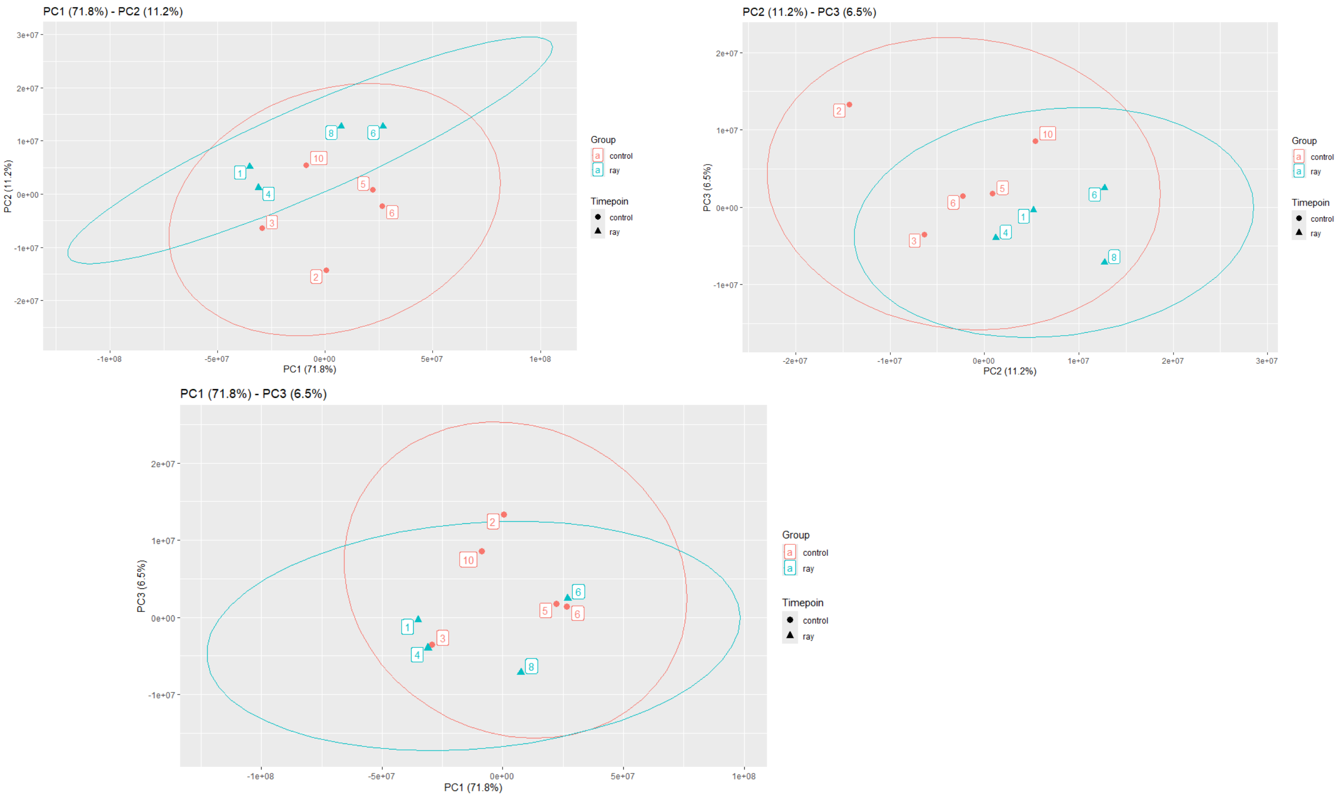

As the data show, the groups are poorly differentiated from one another.

Mean NMR spectra for the study groups were obtained by summing the individual processed spectra for a given group and then dividing the resulting sum by the number of animals (Figure S3). The difference spectrum was obtained by subtraction of mean for control group spectrum from mean spectrum for irradiated group. (Larkin et al., Clin Cancer Res 2022).

Figure S3.

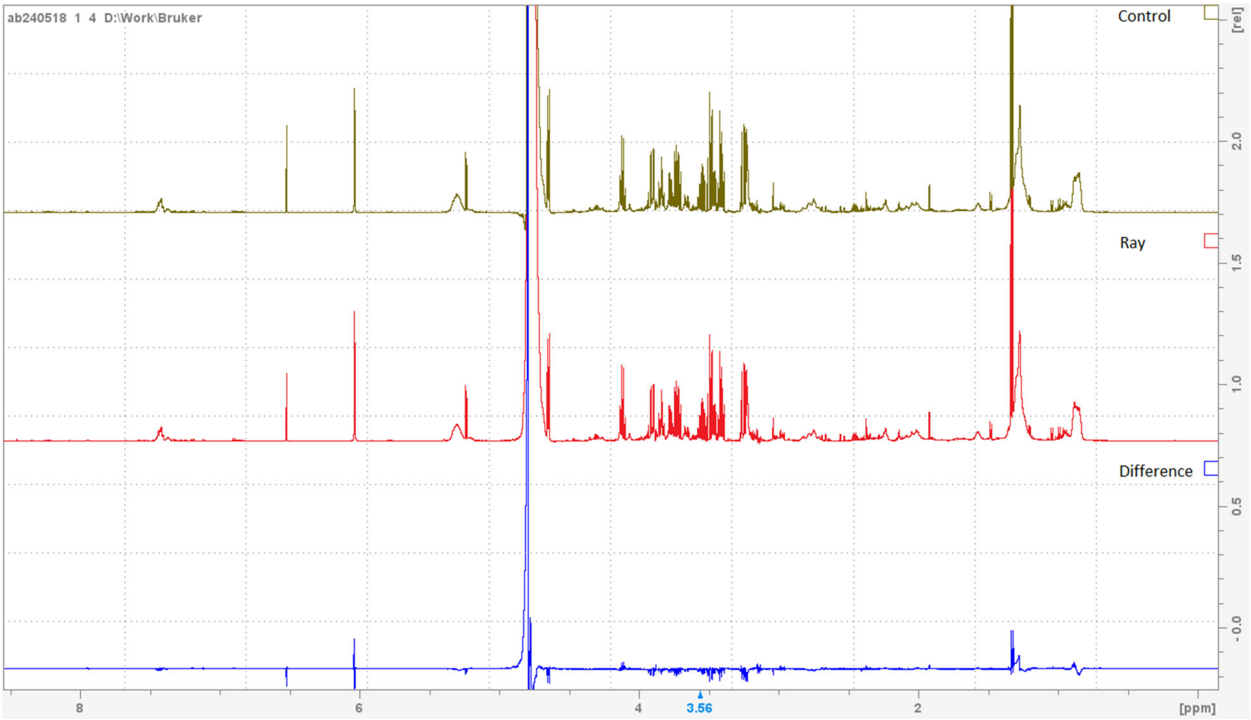


Metabolites undergoing changes were identified based on spectral differences. Metabolite signals in the averaged spectra were integrated; the irradiated group was then compared to the control group to determine the percentage change in metabolite levels in the irradiated group relative to the control. A heat map was generated.
